# Beta-defensin 1, aryl hydrocarbon receptor and plasma kynurenine in major depressive disorder: metabolomics-informed genomics

**DOI:** 10.1038/s41398-017-0056-8

**Published:** 2018-01-10

**Authors:** Duan Liu, Balmiki Ray, Drew R. Neavin, Jiabin Zhang, Arjun P. Athreya, Joanna M. Biernacka, William V. Bobo, Daniel K. Hall-Flavin, Michelle K. Skime, Hongjie Zhu, Gregory D. Jenkins, Anthony Batzler, Krishna R. Kalari, Felix Boakye-Agyeman, Wayne R. Matson, Swati S. Bhasin, Taisei Mushiroda, Yusuke Nakamura, Michiaki Kubo, Ravishankar K. Iyer, Liewei Wang, Mark A. Frye, Rima Kaddurah-Daouk, Richard M. Weinshilboum

**Affiliations:** 1 Department of Molecular Pharmacology and Experimental Therapeutics, Mayo Clinic, Rochester, MN USA; 2 Department of Molecular Pharmacology and Experimental Therapeutics, Mayo Clinic Graduate School of Biomedical Sciences, School of Medicine, Mayo Clinic, Rochester, MN USA; 3 Department of Electrical and Computer Engineering, University of Illinois at Urbana-Champaign, Urbana, IL USA; 4 Department of Health Sciences Research, Mayo Clinic, Rochester, MN USA; 5 Department of Psychiatry and Psychology, Mayo Clinic, Rochester, MN USA; 6 Department of Psychiatry and Behavioral Medicine, Duke University, Durham, NC USA; 7 Department of Medicine, Duke University, Durham, NC USA; 8 Duke Institute for Brain Sciences, Duke University, Durham, NC USA; 9 Department of Systems Biochemistry, Bedford VA Medical Center, Bedford, MA UK; 10 RIKEN Center for Integrative Medical Sciences, Yokohama, Japan; 11 Departments of Medicine and Surgery, University of Chicago, Chicago, IL USA; 12 Present Address: Current affiliation: Assurex Health Inc, Mason, OH USA; 13 Present Address: Current affiliation: PreventionGenetics LLC, Marshfield, WI USA; 14 Present Address: Current affiliation: Department of Pharmacometrics, Duke Clinical Research Institute, Durham, NC USA; 15 Present Address: Current affiliation: Ixcela, Inc, Bedford, MA UK

## Abstract

Major depressive disorder (MDD) is a heterogeneous disease. Efforts to identify biomarkers for sub-classifying MDD and antidepressant therapy by genome-wide association studies (GWAS) alone have generally yielded disappointing results. We applied a metabolomics-informed genomic research strategy to study the contribution of genetic variation to MDD pathophysiology by assaying 31 metabolites, including compounds from the tryptophan, tyrosine, and purine pathways, in plasma samples from 290 MDD patients. Associations of metabolite concentrations with depressive symptoms were determined, followed by GWAS for selected metabolites and functional validation studies of the genes identified. Kynurenine (KYN), the baseline plasma metabolite that was most highly associated with depressive symptoms, was negatively correlated with severity of those symptoms. GWAS for baseline plasma KYN concentrations identified SNPs across the beta-defensin 1 (*DEFB1*) and aryl hydrocarbon receptor (*AHR*) genes that were cis-expression quantitative trait loci (eQTLs) for *DEFB1* and *AHR* mRNA expression, respectively. Furthermore, the *DEFB1* locus was associated with severity of MDD symptoms in a larger cohort of 803 MDD patients. Functional studies demonstrated that DEFB1 could neutralize lipopolysaccharide-stimulated expression of KYN-biosynthesizing enzymes in monocytic cells, resulting in altered KYN concentrations in the culture media. In addition, we demonstrated that AHR was involved in regulating the expression of enzymes in the KYN pathway and altered KYN biosynthesis in cell lines of hepatocyte and astrocyte origin. In conclusion, these studies identified SNPs that were cis-eQTLs for *DEFB1* and *AHR* and, which were associated with variation in plasma KYN concentrations that were related to severity of MDD symptoms.

## Introduction

Major depressive disorder (MDD) is a common, life-threatening psychiatric disease worldwide^1,2^. However, the pathophysiology of MDD is not fully understood. Deficiency of the neurotransmitter serotonin (5-HT) appears to play a role in the pathophysiology of MDD and, as a result, drugs, such as selective serotonin reuptake inhibitors (SSRIs) that enhance serotonergic neurotransmission are used to treat MDD. SSRIs are the standard of care drug therapy for MDD. However, many MDD patients fail to respond to SSRI therapy and response may be delayed for weeks or months^3^. Several genome-wide association studies (GWAS) for both MDD risk^4–6^ and for SSRI treatment outcomes have been performed^7–12^, but few of the top signals from those GWAS have been replicated or functionally validated. This may result, in part, from phenotypic heterogeneity for this disease and/or the lack of biologically based phenotypes^13^.

To advance our understanding of MDD pathophysiology, the Mayo Clinic Pharmacogenomics Research Network-Antidepressant Medication Pharmacogenomics Study (PGRN-AMPS) performed an SSRI trial in which MDD patients were treated with citalopram or escitalopram, with clinical evaluation and blood sampling at baseline and after 4 and 8 weeks of SSRI therapy^14^. GWAS for response^10^ and for plasma drug and drug metabolite levels^15^ in this trial have been published. In an effort to move beyond genomics alone, we have now applied a metabolomics-informed genomic research strategy in which plasma samples from MDD patients were assayed using a “targeted” metabolomic platform that focused on metabolites that might potentially be related to MDD risk and/or response to SSRI therapy. Those metabolites were often related to monoamine neurotransmitters or their metabolites. We then used the metabolomics data to “inform” subsequent genomic analyses, with the goal of discovering mechanisms related to variation in disease risk and/or drug response^16,17^. Previous metabolomic studies in depression and SSRI treatment response have revealed novel signatures^18–20^, and by using a metabolomic-informed genomic research strategy, we had previously identified genome-wide significant single-nucleotide polymorphism (SNP) signals that were associated with plasma 5-HT concentrations and SSRI treatment outcomes, with replication in other SSRI GWAS^21^. However, whether any metabolite(s) or SNP genotype(s) might be associated with the baseline severity of MDD symptoms has not been determined.

The pathophysiology of MDD is almost certainly heterogeneous. Stratification of MDD patients may help identify novel mechanisms that contribute to this disease and could potentially lead to new therapeutic interventions. Extensive data support the hypothesis that variation in monoamine neurotransmission contributes to MDD pathophysiology. In addition, inflammation may also play an important role in MDD pathophysiology^22^. Concentrations of pro-inflammatory cytokines have been shown to be increased in MDD patients as compared with non-depressed patients^23,24^ and have been positively correlated with depressive symptom severity^25,26^. In addition, infection, exposure to endotoxin, therapeutic use of cytokines and psychological stress can all trigger inflammation and lead to depressive symptoms^22,27^. Gut microbe-associated release of inflammatory cytokines may also be a mechanism that can result in depressive symptoms through the “microbiota–gut–brain” axis^28–30^. Importantly, MDD patients with high levels of inflammation have demonstrated poor response to conventional antidepressant therapies, such as SSRIs, but they may respond to cytokine antagonism^31–33^.

**Fig. 1:**
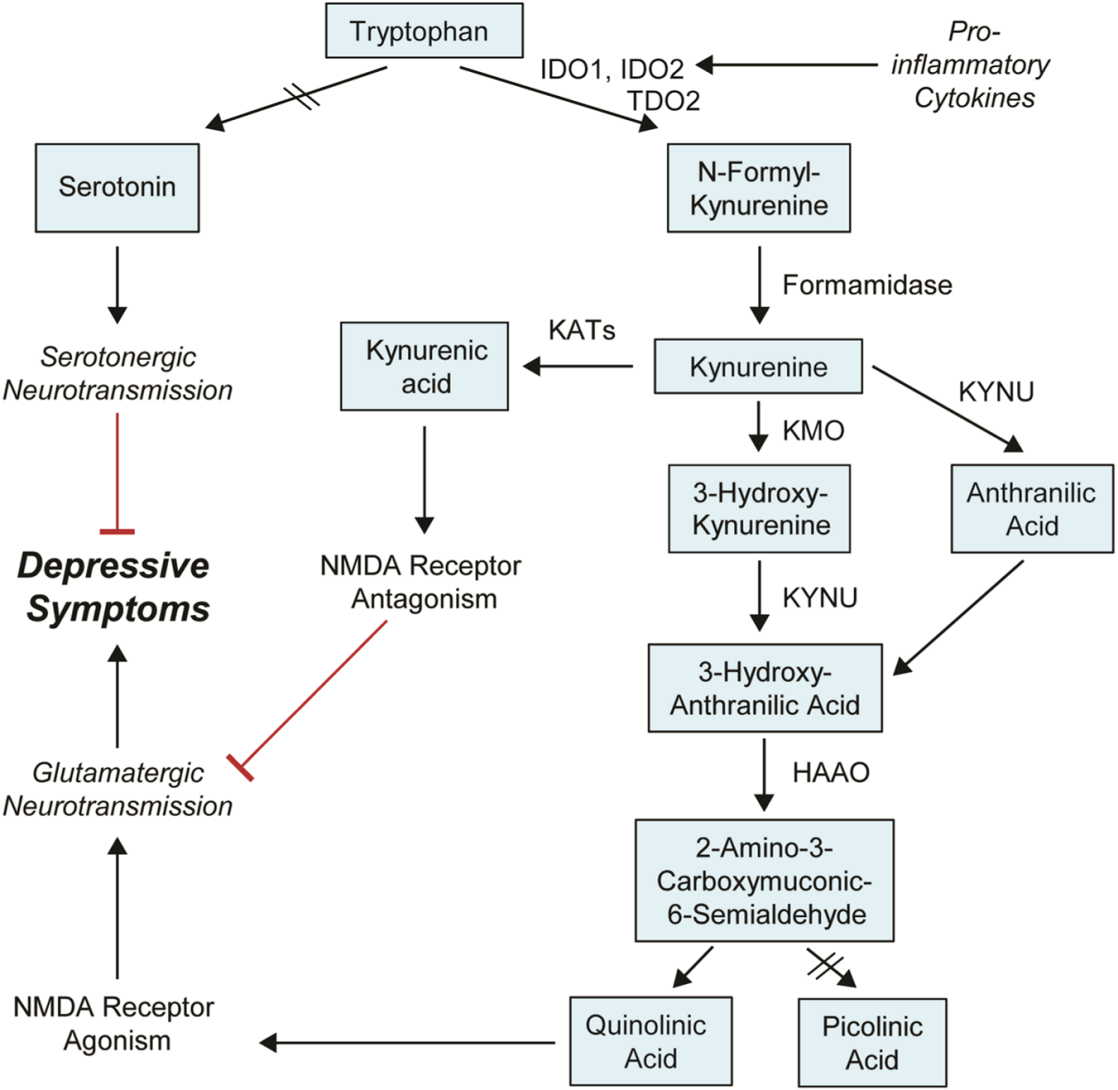
Tryptophan metabolism, kynurenine pathway and major depressive disorder Tryptophan (TRP) is metabolized by two major pathways: the “kynurenine (KYN) pathway” and the “serotonin (5-HT)” pathways. 5-HT cannot pass the blood–brain barrier (BBB). The majority of TRP is metabolized to form KYN in the liver and is released into peripheral blood. The initial and rate-limiting step in the KYN pathway is catalyzed by indoleamine 2,3-dioxygenase 1 (IDO1), IDO2 and/or tryptophan 2,3-dioxygenase (TDO2)—depending on the tissue involved—to form N-formyl-kynurenine. N-formyl-kynurenine is metabolized by formamidase to produce KYN, which can cross the BBB. Approximately 60% of KYN in the CNS originates from the liver. In the periphery and in the CNS, KYN can be further metabolized by either kynurenine aminotransferases (KATs) or by kynurenine 3-monooxygenase (KMO) and kynureninase (KYNU), leading to the generation of the neuroactive metabolites kynurenic acid (KYNA) or quinolinic acid (QUIN), respectively. KYNA, which is neuroprotective, and QUIN, which is neuroactive in the CNS, have opposite effects on the NMDA receptor. Four KATs, encoded by *AADAT*, *CCBL1, CCBL2*, and *GOT2*, have been shown to catalyze the conversion of KYN to KYNA. The other branches of the KYN pathway involve KMO and KYNU, which catalyze the metabolism of kynurenine to form 3-hydroxykynurenine and 3-hydroxyanthranilic acid, respectively. KYNU can also metabolize KYN to form anthranilic acid, which can then be coverted to 3-hydroxyanthranilic acid by nonspecific hydroxylation. 3-Hydroxyanthranilic acid is metabolized further by 3-hydroxyanthranilic acid 3,4-dioxygenase (HAAO) to form either QUIN or, after a series of reactions, picolinic acid

In addition, the “neurotransmission” and the “inflammation” hypotheses are not mutually exclusive and might be linked by tryptophan (TRP) metabolism. TRP is the precursor for both 5-HT and KYN (Fig. 1). Given the importance of 5-HT neurotransmission, the shunting of TRP metabolism from 5-HT to the KYN biosynthetic pathway might contribute to the pathophysiology of depression^34,35^. The first step in the “KYN Pathway” (Fig. 1) is catalyzed by indoleamine 2,3-dioxygenase 1 (IDO1) and IDO2 and/or tryptophan 2,3-dioxygenase 2 (TDO2)^36^. Inflammatory cytokines can increase IDO expression, which leads to activation of the “KYN Pathway” and results in depressive-like behavior in mice^37,38^. However, underlying mechanism(s) remain unclear^39,40^. KYN, unlike 5-HT, can cross the blood–brain barrier, and the majority of KYN in the brain originates from peripheral blood^36^. KYN is then further metabolized by either kynurenine aminotransferases (KATs) or kynurenine 3-monooxygenase (KMO) and kynureninase (KYNU), resulting in the generation of “downstream” metabolites, such as kynurenic acid (KYNA), which is neuroprotective, and quinolinic acid (QUIN), which is neurotoxic^36^. These “downstream” metabolites could play a role in disease pathophysiology through their effects on the N-methyl-D-aspartate (NMDA) receptor^36^ (Fig. 1). Activation of microgila by immune stimuli accelerates the biosynthesis of neurotoxic QUIN and raises the QUIN/KYNA ratio, which has been suggested as a mechanism that might contribute to depression symptoms^36^. Increased QUIN concentrations in cerebrospinal fluid (CSF) have been associated with the development of depressive symptoms in patients after interferon treatment, but no differences in QUIN/KYNA ratios were found^39^. Therefore, even though activation of the KYN pathway has been associated with depression and depressive-like behavior, the cause and effect relationship between the KYN pathway and depression requires clarification, and underlying molecular mechanisms remain unclear.

In the present study, we have applied a metabolomics-informed genomic research strategy to study the severity of MDD symptoms. This approach began with the association of metabolite concentrations with disease severity, followed by GWAS using those metabolites as phenotypes to identify genetic loci associated with variation in concentrations of the metabolites. Of the 31 metabolites that we assayed, plasma KYN concentration was most significantly associated with disease severity in MDD patients prior to antidepressant therapy. GWAS for plasma KYN concentrations identified SNP signals across the beta-defensin 1 (*DEFB1*) and aryl hydrocarbon receptor (*AHR*) genes. DEFB1 is an antimicrobial peptide that is secreted from epithelial cells at the surface of multiple tissues, such as the gastrointestinal (GI) and urinary tracts, and which plays an important role in host defense against microbial infections^41–43^. AHR, a ligand-activated transcription factor that is best known for its role in response to environmental toxins^44,45^, has been reported to regulate the expression of *IDO1/2* ^46–48^, and KYN is an endogenous AHR ligand^49,50^. We then performed DEFB1 and AHR functional validation to determine how variation in the expression of those genes might influence phenotypes—e.g. biochemical pathways—related to KYN in cell lines, including cell lines of immune system, liver and central nervous system (CNS) origin.

## Materials and methods

### Subjects, samples and metabolomic profiling

Patient selection, symptomatic evaluation, and blood sample collection for the PGRN-AMPS clinical trial, which recruited a total of 803 MDD patients, have been described elsewhere^10,14,15,21^. Patients were required to have a baseline Hamilton Depression Rating Scale (HAMD-17) score >14^14^. Plasma samples from 306 randomly selected patients for whom samples at all three time points were available were used to perform metabolomic assays with a quantitative, targeted liquid chromatography electrochemical coulometric array (LCECA) platform. We previously used this dataset to perform our studies of plasma 5-HT in these same patients^21^. See Supplementary Methods for details.

### Correlation of metabolites with disease symptoms

Associations of baseline plasma metabolite concentrations with HAMD-17 scores were assessed using Spearman (partial) correlations. All associations with baseline metabolite levels were adjusted for age and sex. Of the 306 subjects for whom metabolite concentrations were measured, six non-Caucasian and 10 non-compliant patients (based on blood drug assays) were removed from the analysis. Therefore, 290 subjects who had plasma metabolite data were included in the present correlation and GWAS analyses.

### GWAS analyses

GWAS genotyping and imputation using “1000 Genomes” data as reference have been described previously^10,15^. GWAS for baseline plasma KYN concentrations and KYN/TRP (K/T) ratio were performed using linear regression assuming additive allelic effects. See Supplementary Methods for details.

### Expression quantitative trait loci (eQTL) analyses for *DEFB1* and *AHR* SNPs

*DEFB1* and *AHR* expression by genotype were determined using the GTEx dataset^51^. We also used eQTL data from the “BRAINEAC” dataset^52^ to check the *DEFB1* and *AHR* expression in brain tissue. Differences in *DEFB1* and *AHR* expression levels were considered significant when *P*-values were ≤0.05.

### DEFB1–lipopolysaccharide (LPS) neutralization and KYN biosynthesis in monocytic cells

LPS (Sigma, St. Louis, MO) was pre-incubated with or without recombinant human DEFB1 protein (Creative Biolabs, Shirley, NY) in water with 1 mM of dithiothreitol at 37 °C for 30 min. The DEFB1–LPS mixture was then added to serum-free culture media for THP-1 cells (ATCC, Manassas, VA). Cells were pelleted by centrifugation at 4 °C at 100 × *g* for 5 min for extraction of total RNA or protein lysate preparation. The culture media was collected for quantification of KYN and TRP using high-performance liquid chromatography (HPLC) with UV detection. See Supplementary Methods for details.

### AHR and KYN pathways in hepatocyte and CNS-derived cells

HepaRG cells (Biopredict, Rennes, France) were cultured and differentiated into hepatocyte-like cells using the manufacture’s protocol. Differentiated HepaRG cells and human U-87 MG glioblastoma cells (ATCC, Manassas, VA) were transfected with pooled mRNA-specific siRNAs for knockdown (KD) studies. Total RNA and whole cell lysates were then prepared for mRNA quantification and Western blot analysis. Culture media was used for HPLC analyses of KYN and TRP. See Supplementary Methods for details.

### Statistical analyses

Data analyses were performed using JMP (SAS Institute, Cary, NC), and graphs were plotted using GraphPad Prism (GraphPad Software, La Jolla, CA). Statistical comparisons for the functional studies were made using Student’s *t*-test or one-way ANOVA.

## Results

### Association of plasma metabolite concentrations with depressive symptoms

Baseline plasma KYN was the metabolite that was most significantly associated with the baseline severity of depressive symptoms, as measured by the HAMD-17 (*P* = 0.008) (Supplementary Table S1). The “nominal” *P*-values listed in the table have not been corrected for multiple comparisons because our purpose was to identify metabolites for use in GWAS to discover genetic variation associated with variation in metabolite concentrations. The negative correlation coefficient (*r* = −0.157) indicated that lower plasma KYN concentrations were associated with more severe depressive symptoms. To pursue these observations, we performed a discovery GWAS using baseline plasma KYN concentration as the phenotype.

### Plasma KYN GWAS

**Fig. 2:**
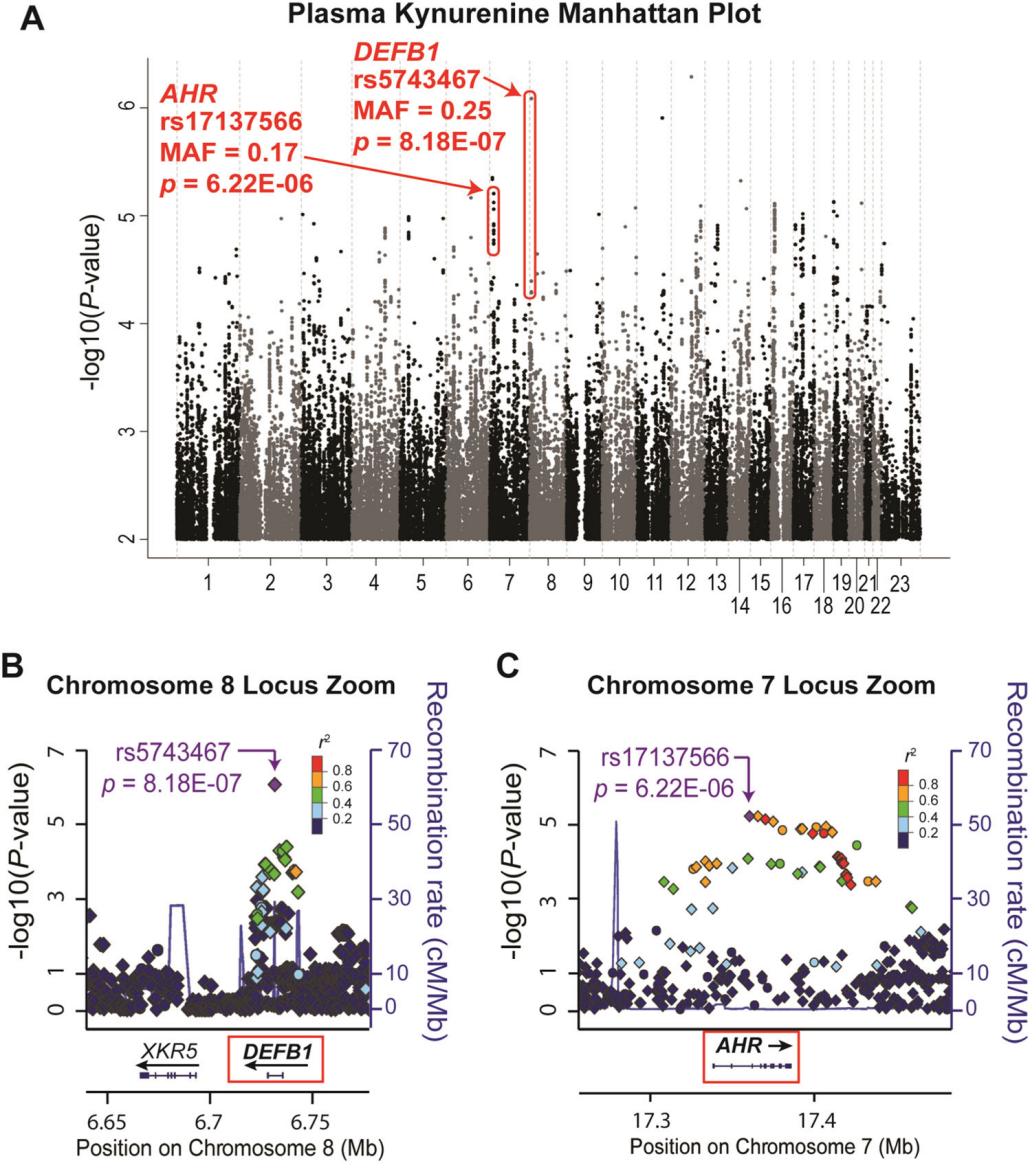
Plasma KYN concentrations GWAS **a** Manhattan plot for baseline plasma KYN concentrations. SNPs across the *DEFB1* and *AHR* genes have been highlighted, with rs5743467 as the “top” *DEFB1* SNP (*P*-value = 8.18E−07) and, rs17137566 as the “top” *AHR* SNP (*P*-value = 6.22E−06). Regional association plots (Locus Zooms) for the *DEFB1* gene **B** and the *AHR* gene **C** are also shown. Circles and diamonds represent observed and imputed SNPs, respectively. The color of each SNP represents its’ linkage disequilibrium (LD) with the “top SNP”, which is colored purple

The Manhattan plot of the GWAS for baseline plasma KYN concentrations showed a cluster of SNPs across the *DEFB1* gene (top SNP rs5743467, *P* = 8.18E−07) and another cluster of SNPs across the *AHR* gene (top SNP rs17137566, *P* = 6.22E−06) (Fig. 2), neither of which were genome-wide significant. Other SNPs with *P*-values lower than those for the SNPs across *DEFB1* and *AHR* were either located in “gene deserts” or had very low (≤0.02) minor allele frequencies (MAFs), which increased the possibility that they might be false positive associations (Supplementary Table S2). Plasma KYN concentrations were higher in patients homozygous for the *DEFB1* variant SNP (rs5743467) genotype (G/G), but lower in patients homozygous for the *AHR* variant SNP (rs17137566) genotype (C/C) as compared with the major alleles (Supplementary Fig. S1). A combination of both *AHR* and *DEFB1* SNP genotypes resulted in the most significant differences in KYN concentrations in which patients who were homozygous wild type for the *DEFB1* SNPs (C/C) and homozygous variant for the *AHR* SNPs (C/C) had the lowest KYN concentrations, and vice versa (Supplementary Fig. S1C). *DEFB1* and *AHR* GWAS SNPs with *P*-values < 5.0E−04 are listed in Supplementary Tables S3 and S4, respectively. MAFs for *DEFB1* SNPs ranged from 0.18 to 0.49 and between 0.17 and 0.23 for *AHR* SNPs in the GWAS subjects, all of whom were Caucasian. These MAFs are consistent with those reported by the 1000 Genomes Project.

Since KYN is a metabolite of TRP and since the plasma K/T ratio is commonly used as a marker for inflammation, we asked whether the K/T ratio might also be associated with severity of MDD symptoms. We found that, like baseline KYN, the K/T ratio was also negatively associated with severity of MDD symptoms as measured by the HAMD-17 (*r* = −0.168, *P* = 0.004), indicating that a decrease in K/T ratio was associated with more severe  MDD symptoms. Therefore, we also performed a discovery GWAS for baseline plasma K/T ratio (Supplementary Fig. S2). The same SNP signal across the *DEFB1* gene that was associated with plasma KYN concentrations, was associated with plasma K/T ratio in these MDD patients (top SNP rs5743467, *P* = 2.15E−07) (Supplementary Table S5). Other SNPs that were associated with variation in the K/T ratio had very low MAFs (≤0.04) (Supplementary Table S6). No other SNP signals were present in both the KYN and K/T ratio GWAS with *P*-values less than 1E−04.

As mentioned previously, the goal of this study was to use metabolomics-informed genomics to understand the biology underlying MDD pathophysiology. DEFB1 is an antimicrobial peptide associated with innate immunity^41,42^, and bacterial infection-induced inflammation has been associated with depression^22^. AHR has been shown to regulate KYN biosynthesis^46–48^, and KYN is an AHR ligand^49,50^. Therefore, we pursued both the *DEFB1* and *AHR* SNP signals functionally even though the *P*-values for these signals were not genome-wide significant.

### *DEFB1* and *AHR* SNPs are cis-eQTLs

**Fig. 3:**
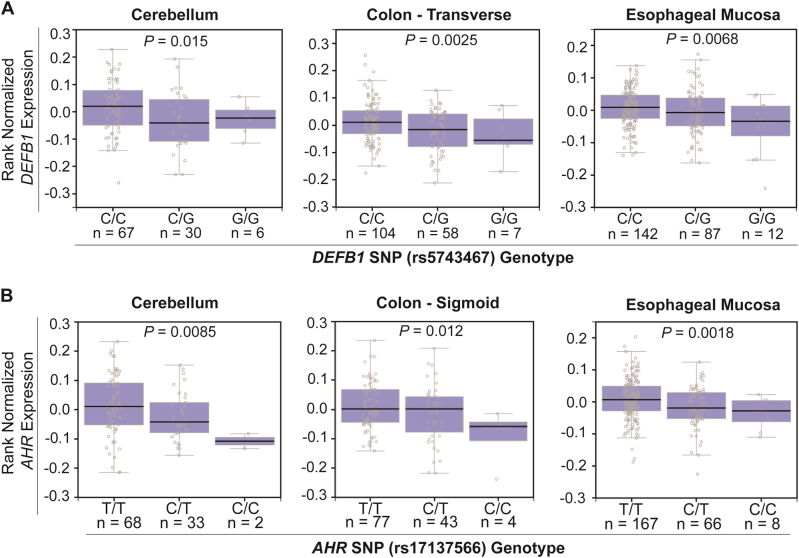
eQTL analysis for the top *DEFB1* **A** and *AHR* **B** SNPs based on the GTEx dataset^51^. **A** *DEFB1* mRNA expression was significantly decreased in human cerebellum (left), transverse colon (middle) and esophageal mucosa (right) from individuals with rs5743467 variant genotypes (G) when compared with WT genotype **(C)** (*P* < 0.05). **B** *AHR* mRNA expression was significantly decreased in human cerebellum (left), sigmoid colon (middle) and esophageal mucosa (right) from subjects with rs17137566 variant genotypes **(C)** when compared with those for subjects homozygous for the WT genotype (T) (*P* < 0.05). mRNA levels were determined by RNA sequencing data available in GTEx

We next determined the relationship of the SNPs identified in the GWAS with *DEFB1* and *AHR* expression using a number of databases. We began with the GTEx dataset^51^ and found that the variant genotype for the *DEFB1* “top SNP”, rs5743467, was associated with decreased *DEFB1* expression, and that the variant genotype for the *AHR* “top SNP”, rs17137566, was associated with decreased *AHR* expression in many tissues, including brain, colon, and esophagus (Fig. 3). We also performed eQTL analyses using the BRAINEAC dataset^52^. The “top SNPs” for *DEFB1* and *AHR* were also cis-eQTLs in human cerebellar cortex, thalamus, and other brain tissues (Supplementary Table S7). The variant alleles were once again associated with decreased *DEFB1* and *AHR* expression. We also used RNA-seq data from 48 lymphoblastoid cell lines (LCLs) generated from European-American subjects to verify these obsersvations^53,54^. DEFB1 was not expressed in this cell line but *AHR* mRNA levels were significantly decreased in LCLs homozygous for variant genotypes for the AHR “top SNP” (rs17137566, *P* = 0.042).

### *DEFB1* and KYN pathway functional genomics in monocytic cells

DEFB1 is an antimicrobial peptide that plays an important role in gut microbiome homeostasis and is constitutively secreted by epithelial cells^41^. Like other antimicrobial peptides, DEFB1 kills bacteria by cell wall permeabilization^41^ and it neutralizes LPS^55^—immune-stimulating molecules on the surface of Gram-negative bacteria—thus protecting the host from bacterial infection and subsequent immune response. Exposure to bacteria or bacterial products such as LPS can activate circulating monocytes, which release pro-inflammatory cytokines that can then activate the KYN pathway by upregulating IDO1 expression^22^. Since infection and inflammation have been associated with increased KYN concentrations and the development of depressive symptoms and since the “microbiota–gut–brain” axis has been hypothesized to play a role in MDD pathophysiology, we investigated the possible influence of DEFB1 on KYN concentrations.

**Fig. 4:**
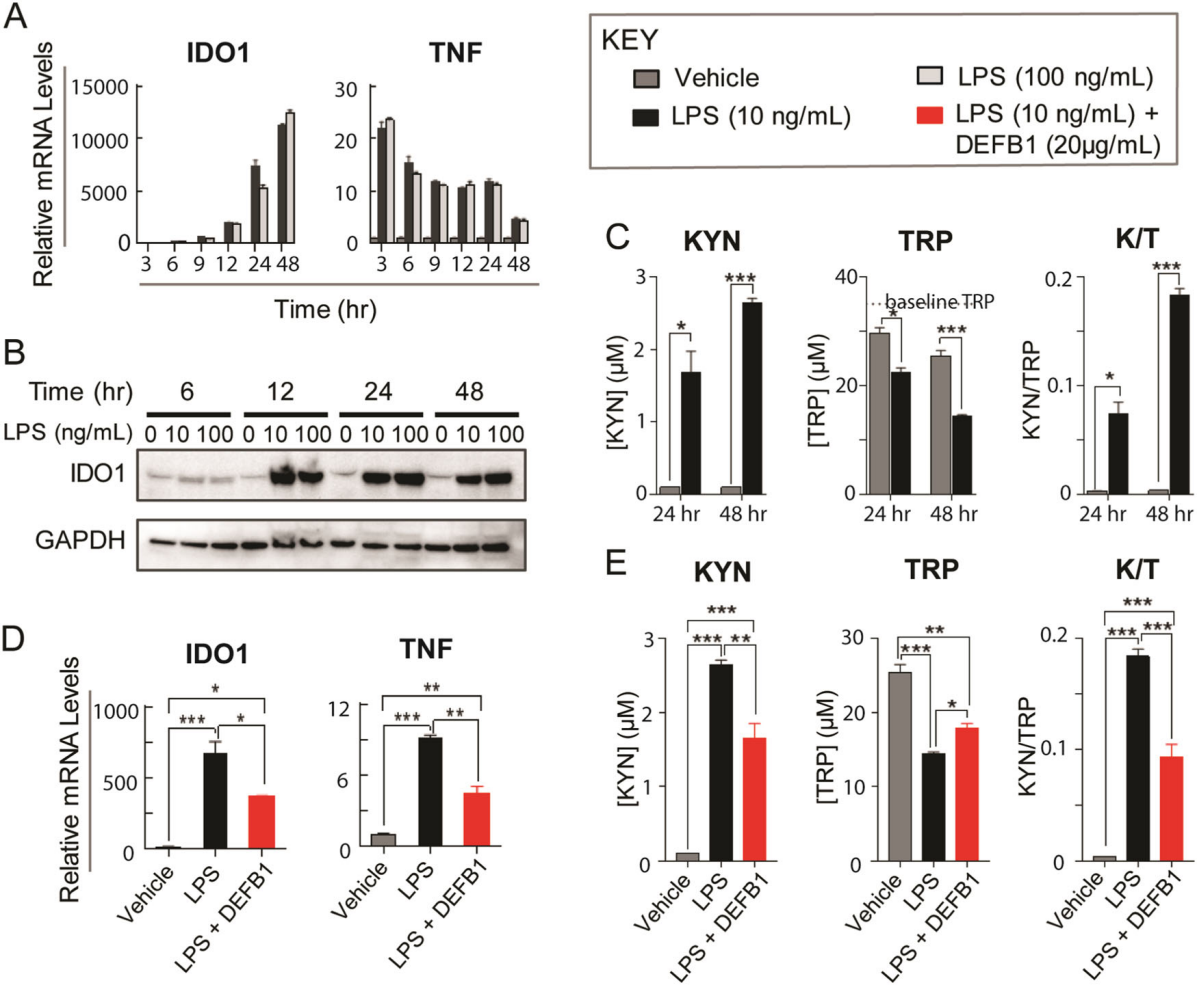
DEFB1 functional studies in THP-1 cells **A** mRNA expression was determined by qRT-PCR after THP-1 cells were exposed to 10 and 100 ng/ml of LPS at different time points. Compared to vehicle treated cells, *IDO1* mRNA levels were significantly increased after LPS treatment. mRNA levels for TNF, a pro-inflammatory cytokine used as a positive control for LPS effect, were also significantly increased after LPS treatment. **B** IDO1 protein expression was increased as analyzed by Western blot after LPS treatment. **C** KYN concentrations (left) in cell culture media were undetectable after 24 and 48 h of vehicle treatments, but were significantly increased after 10 ng/ml LPS treatment. At the same time, TRP concentrations (middle) were significantly decreased in cell culture media after LPS treatment and the K/T ratio was increased (right). After 3, 6, or 12 h of LPS treatment, KYN concentrations were undetectable. **D** mRNA levels for *IDO1* and *TNF* were significantly increased after 10 ng/ml LPS treatment, but recombinant human DEFB1 co-incubation with LPS significantly decreased mRNA levels for *IDO1* and *TNF* when compared with LPS treatment alone. **E** When DEFB1 was co-incubated with LPS as compared to LPS alone, KYN concentrations (left) in cell culture media were significantly decreased, TRP concentrations were increased (middle) and K/T ratios (right) were decreased after DEFB1 was pre-incubated with LPS when compared with results for cells treated with LPS alone. *N* ≥ 3 for all the experiments. Data = mean ± SEM, with statistical significance determined by two-tailed *t*-test denoted as *P < 0.05, **P < 0.01, and ***P < 0.001

Specifically, we used THP-1 cells, a widely used human monocytic cell line^56–58^, exposed to LPS to mimic the “innate immune response” triggered by bacterial  infection or exposure to bacterially derived compounds, such as LPS. The mRNA expression of the KYN biosynthesizing enzyme IDO1 was significantly increased after LPS treatment (see Figs. 1 and 4A). Tumor necrosis factor alpha (TNF) mRNA was also highly induced after LPS exposure. We used TNF expression as an indicator of LPS stimulation. The level of IDO1 protein was also significantly induced after LPS treatment (Fig. 4B). The induction of IDO1 was associated with an increase in KYN concentration, a decrease in TRP concentration and—as expected—an increased K/T ratio in the cell culture media (Fig. 4C). We also observed that the expression of TDO2 and IDO2 mRNA was induced after LPS treatment (data not shown). However, TDO2 protein was undetectable, perhaps because the level of TDO2 expression in this cell line is very low, suggesting a limited contribution to KYN synthesis in this cell line. IDO2 mRNA was also much less highly induced than that of IDO1. Therefore, immune stimulation, as modeled by LPS treatment, altered KYN pathway metabolism in THP-1 monocytic cells primarily by the induction of IDO1 expression.

We then used THP-1 cells to investigate the effect of DEFB1 on LPS-stimulated KYN metabolism. Addition of recombinant DEFB1 to THP-1 cell culture media decreased LPS response as measured by TNF expression. In addition, IDO1 expression was decreased after the addition of DEFB1, with a parallel decrease in KYN concentration, an increase in TRP concentration and a decrease in the K/T ratio as compared to LPS treatment alone (Fig. 4D, E). Therefore, DEFB1 could alter the response of THP-1 cells to LPS treatment, as previously described^55^, and it could alter KYN biosynthesis. These results were consistent with our GWAS data in which the variant genotype (G/G) for the *DEFB1* “top SNP” was associated with decreased DEFB1 expression (Fig. 3A), higher plasma KYN concentrations (Supplementary Fig. S1A and S1C) and a higher K/T ratio in MDD patients (Supplementary Table S5).

### *AHR* and KYN pathway functional genomics in hepatic-derived and astrocytic-derived cells

**Fig. 5:**
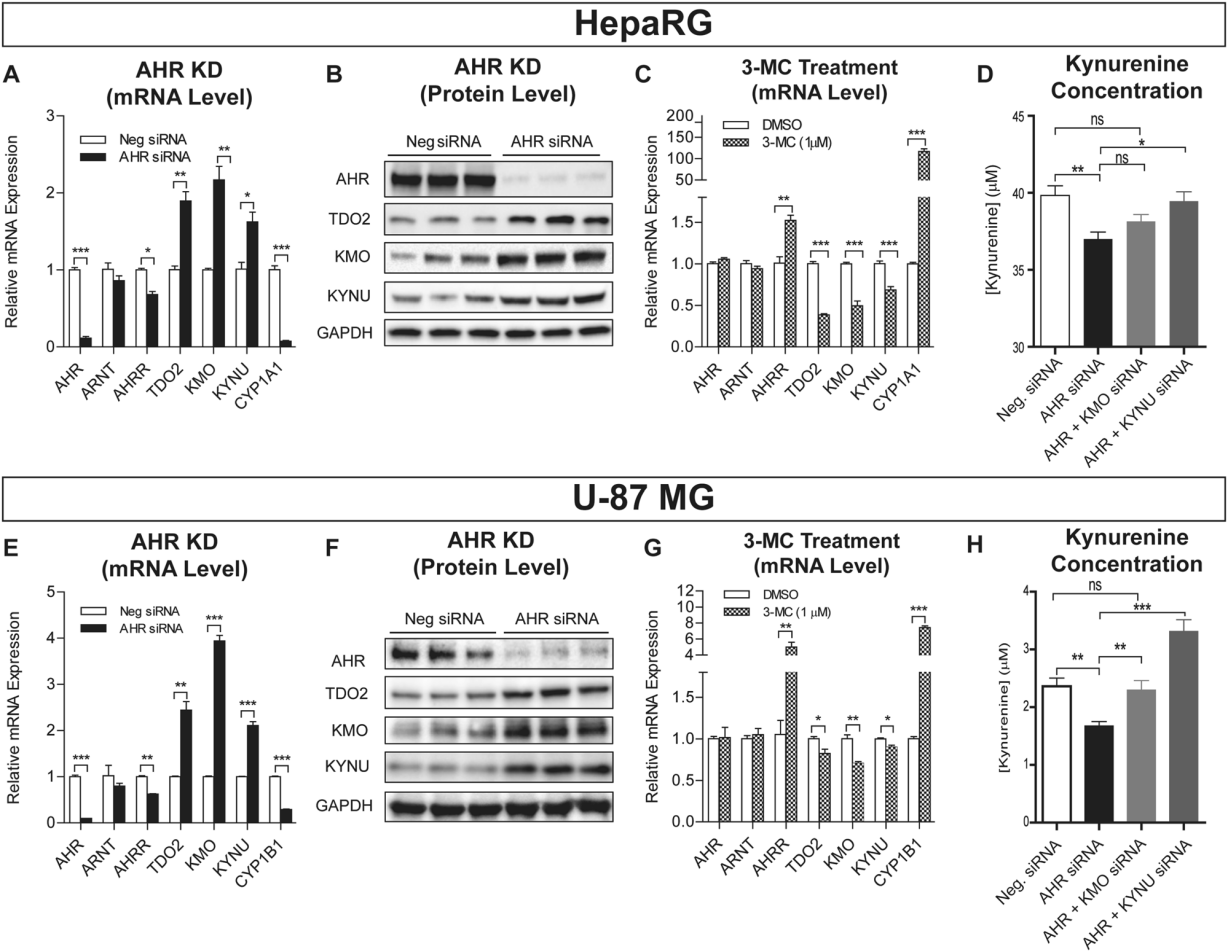
AHR functional studies in HepaRG and U-87 MG cells **A** mRNA expression determined by qRT-PCR after *AHR* KD in HepaRG cells. *TDO2*, *KMO*, and *KYNU* expression was significantly increased and *AHRR* and *CYP1A1* expression was significantly decreased following *AHR* KD. **B** Protein expression analysis for AHR, TDO2, KMO, and KYNU by Western blot analysis after *AHR* KD in HepaRG cells. **C** mRNA expression in HepaRG cells determined by qRT-PCR after 24-h treatment with 1 µM 3-MC, an AHR agonist. *TDO2*, *KMO*, and *KYNU* expression was significantly decreased. *AHRR*, *CYP1A1* (in HepaRG), was  induced by 3-MC, indicating that AHR was activated by the treatment. **D** KYN concentrations in HepaRG cell culture media after AHR and AHR plus KMO or KYNU KD. **E** *TDO2*, *KMO*, and *KYNU* expression was significantly increased while *AHRR* and *CYP1B1* expression was significantly decreased following *AHR* KD. **F** AHR, TDO2, KMO, and KYNU protein concentrations were significantly altered in U-87 MG cells after AHR KD. **G** mRNA expression in U-87 MG cells after 1 µM 3-MC treatment showed significant decreases in *TDO2*, *KMO*, and *KYNU* expression and significant increases in *AHRR* and *CYP1B1* expression. **H** KYN concentrations in U-87 MG cell culture media were significantly decreased after AHR KD but were less decreased after AHR KD together with KMO or KYNU KD. *N* ≥ 3 for all experiments. Data = mean ± SEM, with statistical significance determined by two-tailed *t*-test denoted as *P < 0.05, **P < 0.01 and ***P < 0.001 when compared with control. ns = not significant

The majority of plasma KYN is synthesized in the liver where TDO2 is highly expressed, and peripheral KYN is the source of ∼60% of KYN in the brain^36^. Therefore, we also performed functional studies with HepaRG cells—liver progenitor cells that can be differentiated into hepatocyte-like cells that retain many of the characteristics of human hepatocytes^59^. To mimic the effect of the variant *AHR* SNPs that were associated with decreased AHR expression (Fig. 3B), we knocked down AHR in HepaRG cells and assayed the mRNA expression of enzymes, such as TDO2, IDO1, IDO2, KMO, KYNU, CCBL1, AADAT, CCBL2, GOT2, and HAAO that regulate KYN pathway metabolism (see Fig. 1). We also determined the expression of *CYP1A1* as a prototypic gene that is regulated by AHR^60^. As anticipated, KD of AHR dramatically decreased CYP1A1 expression (Fig. 5A). AHR KD also resulted in significantly increased mRNA expression for TDO2, the gene that encodes the rate-limiting enzyme for KYN biosynthesis in liver^36^, as well as KMO and KYNU, genes encoding “downstream” KYN pathway enzymes that catalyze reactions leading to the generation of QUIN—a neurotoxic NMDA receptor agonist (Fig. 1). IDO1 is not expressed in this cell line, and mRNA levels for other KYN pathway genes that we tested were unchanged after AHR KD. The increase in TDO2, KMO, and KYNU mRNA expression was also reflected in protein levels as measured by Western blot analysis (Fig. 5B). After treatment with a prototypic AHR ligand, 3-methylcholanthrene (3MC)^61^, expression of TDO2, KMO, and KYNU was significantly decreased (Fig. 5C), while the expression of CYP1A1, as anticipated, increased.

To determine the relationship between these results and KYN biosynthesis, concentrations of KYN and TRP were assayed in the HepaRG culture media before and after AHR KD. KYN concentrations in the cell culture media decreased significantly after AHR KD when compared with cells exposed to control siRNA (Fig. 5D). This occurred in spite of induction of the expression of TDO2, the enzyme that catalyzes the rate-limiting step in the biosynthesis of KYN in liver, probably as a result of increased expression of KMO and KYNU—“downstream” enzymes that utilize KYN as a substrate. In support of that hypothesis, KD of AHR together with KD of either KMO or KYNU resulted in increased KYN culture media concentrations when compared with AHR KD alone (Fig. 5D). Meanwhile, media TRP concentrations decreased both after AHR KD and AHR KD together with KMO or KYNU KD (Supplementary Fig. S3B). Although KYN concentration did not display a statistically significant change after AHR + KMO KD vs. AHR KD alone, there was a trend toward KYN concentration increase after AHR + KMO KD. Finally, after AHR KD combined with exposure to a potent KMO inhibitor (Ro 61-8048, IC50 = 0.037 µM), KYN concentrations in the cell culture media increased although TRP concentrations remained decreased, further supporting the conclusion that increased KMO expression after AHR KD contributed to the observed decrease in KYN media concentrations (Supplementary Fig. S3C).

These results showed that AHR KD, a model that we had used to mimic the effect of variant *AHR* SNP genotypes on AHR expression, resulted in decreased KYN concentrations in cell culture media. This result was consistent with our GWAS, which showed that variant *AHR* SNP genotypes (C/C) were associated with lower AHR mRNA (Fig. 3B) and with lower levels of plasma KYN in MDD patients (Supplementary Fig. S1B).

KYN biosynthesis in brain primarily occurs in astrocytes^36^. To determine whether the regulation of KYN pathway genes by AHR might also occur in CNS-derived cells, we used an astrocyte-derived cell line, the U-87 MG glioblastoma cells, to perform experiments parallel to those performed with HepaRG cells. U-87 MG glioblastoma cells express TDO2 as well as KYNU while expressing KMO at a relatively low level. We found that AHR KD significantly increased TDO2, KMO, and KYNU expression at both the mRNA and protein levels (Figs. 5E, F) and that 3-MC treatment down-regulated TDO2, KMO, and KYNU expression (Fig. 5G), similar to the HepaRG results (Fig. 5A-D). We also measured KYN and TRP concentrations in U-87 MG culture media. Consistent with the HepaRG results, KYN concentrations in U-87 MG cell culture media were significantly decreased following AHR KD but rose when KMO or KYNU were knocked-down together with AHR (Fig. 5H), indicating that increased expression of KMO and KYNU after AHR KD plays a role in the decrease in KYN concentration after AHR KD alone. We observed that the increase in KYN concentration after AHR KD together with KYNU KD was more significant than that for KMO KD (Fig. 5H), which might indicate that KYNU KD affects two KYN downstream metabolizing pathways (Fig. 1). We also observed that culture media TRP concentrations decreased after AHR KD but that treatment with the KMO inhibitor increased KYN media concentrations after AHR KD, with continued decrease in TRP concentrations, supporting the conclusion that increased KMO expression after AHR KD is one cause of the decrease in KYN concentrations (Supplementary Fig. S3E–H). Taken together, these results strongly suggest that the decrease in KYN concentration after AHR KD resulted from enhanced KYN downstream metabolism as a result of increased expression of KMO and KYNU.

Finally, although TRP catabolism in the brain occurs mainly in glial cells, we also tested the effect of AHR KD using neurons differentiated from human-induced pluripotent stem cells (iPSCs). TDO2, KMO, and KYNU mRNA levels also increased after AHR KD in these neuronal cells (Supplementary Fig. S4). Baseline levels of expression for these genes were low in the iPSC-derived neurons, which resulted in low KYN concentrations (0.23 + 0.02 µM) in the culture media, perhaps explaining why we did not observe significant changes in these concentrations after *AHR* KD (data not shown).

### Association of *DEFB1* SNP signals with severity of MDD symptoms

To determine whether the *DEFB1* and *AHR* SNPs that were identified during the GWAS for plasma KYN concentrations might be directly associated with severity of MDD symptoms, we determined associations of these SNPs with the severity of symptoms measured by both HAMD-17 and QIDS-C16 scores in the larger cohort of all 803 patients who were recruited to the Mayo-PGRN AMPS study^14^. One of the *DEFB1* SNPs, rs2702877, was significantly associated with the severity of MDD symptom as measured by both HAMD-17 (*P* = 1.74E−04) and QIDS-C16 scores (*P* = 1.25E−05) for all 803 patients (Supplementary Table S8). The variant allele for this SNP was associated with more severe symptoms at baseline. This SNP was associated with increased DEFB1 expression and lower KYN concentrations. None of the *AHR* SNPs was associated with HAMD-17 or QIDS-C16 scores in these 803 patients.

This rs2702877 SNP was not in LD with the rs5743467 “top hit” SNP that was most highly associated with plasma KYN concentrations (*r* ^2^ = 0.09 in Caucasian, 1K Genomes data). However, rs2702877 was a stronger eQTL (Supplementary Fig. S5) and had a higher MAF (0.31) than the rs5743467 SNP.

## Discussion

We have applied a metabolomics-informed genomic research strategy to study the severity of MDD symptoms in an attempt to address the molecular basis for these symptoms. We began by assaying 31 metabolites that might be relevant to MDD pathophysiology, including compounds from the tryptophan, tyrosine, and purine pathways, in plasma samples from 290 MDD patients. Plasma KYN was the metabolite for which concentrations were most highly associated with baseline depressive symptoms as measured by HAMD-17 scores. KYN concentrations were negatively correlated with severity of depressive symptoms (Supplementary Table S1). This result was consistent with a recently published study that reported plasma KYN concentrations were negatively associated with suicidal ideation, one of the most severe symptoms of MDD^62^.

KYN can cross the blood–brain barrier and peripheral KYN, primarily generated in the liver, is the source of ∼60% of CNS KYN^36^. However, the relationship of plasma KYN concentration to MDD symptom severity remains unclear. Previous studies have not consistently determined whether increased or decreased plasma KYN concentrations were associated with symptoms of MDD, perhaps because of the metabolomic assays used, MDD phenotypic heterogeneity, sample size and/or cohort composition^39,63^. In an attempt to further understand possible mechanisms underlying the association of plasma KYN concentrations with severity of MDD symptoms, we performed a GWAS with plasma KYN concentration as the phenotype to identify genetic factors that might contribute to inter-individual variation in plasma KYN concentrations. The GWAS identified SNPs across the *DEFB1* and *AHR* genes that were associated with variation in baseline plasma KYN concentrations in these MDD patients (Fig. 2). The same SNP signal for *DEFB1* was also associated with variation in the plasma K/T ratio (Supplementary Fig. S2). The SNP signals across *DEFB1* and *AHR* were cis-eQTLs for DEFB1 and AHR expression, respectively (Fig. 3).

Depression has been associated with inflammation, which can be influenced by the interaction between the microbiome and the host. DEFB1 is an antimicrobial peptide that plays a role in host defense against bacterial infection. Therefore, we performed functional studies to investigate the possible relationship of DEFB1 to KYN biosynthesis in innate-immune-related cell lines. DEFB1 inhibited LPS-stimulated transcription of KYN pathway genes, which resulted in smaller increases in KYN concentrations in cell culture media after LPS exposure. These results were consistent with our GWAS data indicating that WT genotype for the *DEFB1* “top SNP”, which was associated with increased *DEFB1* expression (Fig. 3), was associated with lower plasma KYN concentrations in MDD patients (Supplementary Fig. S1A).

DEFB1 kills bacteria by the same mechanism as other antimicrobial peptides but, unlike other antimicrobial peptides that are induced by immune stimuli, DEFB1 is constitutively expressed and secreted from epithelial cells^41^. Furthermore, the expression of DEFB1 is not affected by pro-inflammatory or bacterial molecules^43^, such as LPS^64^. DEFB1 is also known to play a role in maintaining gut–microbiome homeostasis^43^.Therefore, intrinsic variation in DEFB1 expression, such as that resulting from genetic polymorphisms, could be related to inter-individual variation in disease phenotypes. In fact, genetic polymorphisms in *DEFB1* have been associated with inflammatory bowel disease^65^ and other immune-related diseases^66–68^. The SNPs across *DEFB1* identified in our GWAS for baseline plasma KYN concentrations and the GWAS for plasma K/T ratio in our 290 MDD patients were strong cis-eQTLs for DEFB1 expression in many tissues, including gut (Fig. 3). One of the *DEFB1* SNPs was also directly associated with the severity of MDD symptoms in all 803 patients who had been recruited to the Mayo-PGRN AMPS trial (Supplementary Table S8). Although broadly expressed in many tissues, DEFB1 appears to be functionally important at mucosal membranes, since its antimicrobial activity can be increased in an anaerobic environment which changes its structure to a more active form by disulfide bond reduction^42^. The *DEFB1* SNPs are not only associated with plasma KYN concentrations but also with plasma KYN/TRP ratios. Maintenance of homeostatic balance among commensal microbes is critical for health^69,70^, and evidence suggests that gut dysbiosis could alter brain function and mental illness, including MDD^28,29^. Therefore, our data suggest that SNPs across *DEFB1* may be associated with individual variation in DEFB1 levels and variation in host–microbiome interaction, which could result in altered KYN biosynthesis and MDD symptom severity. Future studies that assay DEFB1 and inflammatory cytokines in patient samples, as well as studies of MDD patient microbiota will be required to test the hypothesis that DEFB1 may play a role in MMD pathophysiology through the “microbiota–gut–brain” axis.

In addition to the *DEFB1* signal, SNPs across *AHR* were also associated with plasma KYN concentrations in our MDD patients. Since the majority of KYN in the brain originates from the liver, and since AHR has been shown to play a role in KYN biosynthesis by influencing the expression of IDO1, we also investigated the impact of AHR expression on KYN biosynthesis in both hepatocytes and CNS-derived cell lines. We demonstrated that AHR KD resulted in increased expression of TDO2, KMO, and KYNU, enzymes that catalyze KYN biosynthesis and its downstream metabolism (Fig. 1). TDO2 is the major enzyme responsible for the biosynthesis of KYN in the liver^71^. KMO catalyzed biotransformation ultimately results in the conversion of KYN to the neurotoxic metabolite, QUIN, an NMDA receptor agonist, which has been reported to be associated with depressive symptoms^39^ and other neuropsychiatric diseases^72–74^. The increase in KMO and KYNU expression after AHR KD may help explain why variant AHR SNP genotypes, which were associated with lower AHR expression, were also associated with lower plasma KYN concentrations. Since the potential actions of KYN metabolites on the glutamatergic system^75,76^ are thought to contribute to MDD risk, our results suggest that AHR may play a role in regulating KYN biosynthesis and, as a result, may contribute to MDD pathophysiology.

In summary, our results raise the possibility of the involvement of *DEFB1* and *AHR* genetic polymorphisms in MDD pathophysiology—at least in part through an effect on the biosynthesis of KYN.

## Electronic supplementary material

Supplementary Materials
