# Supplementary material for: Beta-defensin 1, aryl hydrocarbon receptor and plasma kynurenine in major depressive disorder: metabolomics-informed genomics
Source: Transl Psychiatry. 2018 Jan 10;8:10. doi: 10.1038/s41398-017-0056-8 (PMC5802574; doi:10.1038/s41398-017-0056-8)
Supplement: Supplementary file 1 — Supplementary Materials [file 41398_2017_56_MOESM1_ESM.docx]

**SUPPLEMENTARY MATERIALS**

***SUPPLEMENTARY METHODS:***

**Subjects and Samples**

Patients were enrolled in the Mayo-PGRN-Antidepressant Medication Pharmacogenomic Study (AMPS) SSRI clinical trial after diagnosis of MDD with a Hamilton Depression Rating Scale (HAMD-17) > 14 without psychosis or mania[^1^](#_ENREF_1). None of the patients in this cohort were known to suffer from other major diseases. Blood samples were collected from patients before the initiation of SSRI therapy. To obtain plasma from EDTA anti-coagulated blood, samples were centrifuged at 8000×g for 20 minutes. The plasma samples were then centrifuged once again at 10,000×g for 10 minutes to ensure complete removal of platelets. The plasma samples were stored at -80°C prior to analysis. These 306 samples were used to perform the metabolomics assays. A portion of the blood sample was also used to extract DNA for genome-wide single nucleotide polymorphism (SNP) genotyping.

**Metabolomic Profiling**

A quantitative targeted Liquid Chromatography Electrochemical Coulometric Array (LCECA) platform that has been extensively validated and used in prior studies[^2^](#_ENREF_2)^,^ [^3^](#_ENREF_3) of neurodegenerative and psychiatric disorders was utilized for metabolomic profiling. In total, 31 metabolites, primarily metabolites in the tryptophan, tyrosine and purine pathways, were assayed in these plasma samples. A full list of the metabolites assayed can be found in **Supplementary Table S1** and in previous publications[^2^](#_ENREF_2)^,^ [^3^](#_ENREF_3).

**GWAS Analyses**

Genotyping of genome-wide SNPs and imputation performed by using “1000 Genomes” data as reference have been described previously[^4^](#_ENREF_4)^,^ [^5^](#_ENREF_5). Genotyping was performed at the RIKEN Center for Integrative Medical Science (Yokohama, Japan) using Illumina Human610-Quad BeadChips (Illumina, San Diego, CA, USA). More than 97% of study participants reported their race/ethnicity as white non-Hispanic. Further evaluation of genetic ancestry by use of SNPs for ancestry clustering using the STRUCTURE software provided estimates of the probability of European ancestry[^4^](#_ENREF_4). Linear regression models were used to assess associations between SNPs and KYN levels and between SNPs and K/T ratios. Plasma KYN concentrations and plasma K/T ratios were transformed using a Van der Waerden rank (normal score) transformation and the analyses were adjusted for age and gender. Approximately 7.5 million imputed and observed SNPs were analyzed using as criteria a minor allele frequency (MAF) ≥ 0.01 (imputed and observed SNPs) and dose *r^2^* ≥0.3 (imputed SNPs only).

**Cell Culture**

THP-1 cells were obtained from the American Type Culture Collection (ATCC) (Manassas, VA). THP-1 cells were grown at 37°C with 5% CO_2_ in RPMI 1640 media (Gibco, Grand Island, NY) supplemented with 10% fetal bovine serum (FBS) (Atlanta Biologicals, Inc., Flowery Branch, GA).

Undifferentiated HepaRG human hepatic cells (HPR101) were obtained from Biopredict (Rennes, France) and were cultured and differentiated into fully functional hepatocyte-like cells according to the manufacture’s protocol. HPR101 cells were grown at 37°C with 5% CO_2_ in William's E media (Gibco, Grand Island, NY) supplemented with 1× GlutaMAX (Gibco, Grand Island, NY) and 10% HepaRG Growth Medium Supplement (Biopredict, Rennes, France) for 10-14 days until confluent. Cells were then switched into HepaRG Differentiation media for 2 weeks. Differentiated HepaRG cells were used directly for transfection.

The human U-87 MG glioblastoma cells were purchased from ATCC. U-87 MG cells were grown at 37°C with 5% CO_2_ in DMEM/F12 media (Gibco, Grand Island, NY) supplemented with 10% FBS (Atlanta Biologicals, Inc., Flowery Branch, GA). 300,000 cells were plated in each well of a 12-well cell culture plate, and were subjected to knockdown (KD) or incubation with 3-methylcholanthrene (3-MC) (Sigma, St. Louis, MI).

Human neural stem cells (hNSC) were purchased from Axol Bioscience (Cat#: ax0016, Cambridge, UK) and were differentiated according to the company’s protocol.

All of those cell lines were kept in early passages in working aliquots that are stored in liquid nitrogen to ensure that the experiments were performed with cells that are within the initial 10 passages.

**DEFB1-Lipopolysaccharide Neutralization and KYN Biosynthesis in THP-1 Cells**

For LPS treatment, LPS from *Escherichia coli* 055:B5 (Product #: L4524, Sigma, St. Louis, MO) were dissolved in sterile distilled water and added into the THP-1 cell cultures at final concentrations of 10 ng/mL or 100 ng/mL. For total RNA extraction, approximately 500,000 THP-1 cells in 1 mL of serum-free media were seeded in each well of a 24-well cell culture plate. Cells were harvested after 3, 6, 9, 12, 24 and 48 hours of LPS treatment. For protein preparation and HPLC analysis, approximately 2,500,000 THP-1 cells in 5 mL of serum-free media were seeded in each well of a 6-well cell culture plate. Cells were harvested after 6, 12, 24 and 48 hours of LPS treatment. After centrifugation at 4°C at 100 ×g for 5 mins, cell pellets were collected for protein lysate preparation and the culture media was collected for HPLC analyses of KYN and TRP.

DEFB1-LPS neutralization assay was modified based on previously published methods[^6^](#_ENREF_6). Recombinant human DEFB1 proteins (Creative Biolabs, Shirley, NY) that were reconstituted in sterile distilled water containing 0.1% BSA to a concentration of 2 mg/mL and 100× LPS (1000 ng/mL) were pre-incubated with 1mM of DTT at 37°C for 30 min. Purity of the recombinant DEFB1 protein was reported to be >98% by SDS-PAGE and HPLC analyses. The same 0.1% BSA buffer without recombinant DEFB1 was pre-incubated with LPS to serve as a control treatment. 10 uL of the pre-incubated DEFB1-LPS mixtures, LPS alone or water were added to each well of a 24-well cell culture plate that incubated 500,000 THP-1 cells in 1 mL of serum-free media. After 12 hours of incubation, cells were harvested for total RNA extraction. Cell culture media was collected after 48 hours of incubation for HPLC analyses of KYN and TRP.

**AHR and KYN Pathways in HepaRG, U-87 MG and Neuronal Cells**

The HepaRG and U-87 MG cells were transfected with pooled siRNAs that targeted human *AHR*, *KMO* or *KYNU* (GE Healthcare, Lafayette, CO) separately by using Lipofectamine RNAiMAX reagent (Life Technologies, Grand Island, NY). The hNSC-derived neuronal cells were transfected with pooled *AHR* siRNA lentivirus that was obtained from Applied Biological Materials Inc. (Richmond, BC, Canada). After 24-hour transfections, cell culture media was replaced with fresh media and the cells were incubated for an additional 48 hours. The cells were then harvested for total RNA and for protein assays. Cell media was also collected from treatment and controls for the measurement of KYN and TRP concentrations. Fresh cell culture media was used as a “blank” control when KYN and TRP concentrations were measured in the cell culture media.

A “prototypic” AHR ligand, 3-MC (Sigma, St. Louis, MI), was used to treat HepaRG and U-87 MG cells to activate AHR signaling in these cells. Control cells were treated with dimethyl sulfoxide (DMSO) which had been used to dissolve 3-MC. Cells were incubated with 1 µM of 3-MC for 24 hours and were then harvested for total RNA extraction.

A KMO inhibitor, Ro 61-8048 (Sigma, St. Louis, MI) was also used to treat HepaRG™ and U-87 MG cells after *AHR* KD. Cells were incubated with 1 µM of Ro 61-8048 which has an IC_50_ value of 0.037 µM[^7^](#_ENREF_7), after 24 hours of *AHR* siRNA transfection. After 48 hours incubation, cells were harvested for total RNA extraction and cell media was collected for KYN and TRP assay.

**mRNA Quantification**

Total RNA was extracted from cells by using the QuickRNA miniPrep kit (Zymo Research, Irvine, CA). mRNA levels were quantified by qRT-PCR using the one step RNA-to-Ct kit (Life Technologies, Grand Island, NY) and PrimeTime^®^ pre-designed qPCR primers (IDT, Inc., Coralville, Iowa). Gene expression analyses were performed using the ΔΔCt method[^8^](#_ENREF_8), and *GAPDH* was used as the internal reference gene.

**Western Blot Analysis**

Total protein was isolated from cells after transfection using M-PER buffer (ThermoFisher, Dubuque, IA). Equal quantities of denatured proteins were loaded onto premade gels (Bio-Rad, Hercules, CA) to separate proteins. Proteins were transferred electrophoretically from the gels onto PVDF membranes that were then blocked with 5% non-fat milk at room temperature for 1 hour. The membranes were then incubated with primary antibodies (detailed information has been listed in **Supplementary Table S9**) dissolved in 5% BSA prepared in TBST at 4°C overnight with gentle rocking. Following incubation, the membranes were washed vigorously several times with TBST and were then incubated with appropriate secondary antibodies dissolved in 5% non-fat milk at room temperature for 1 hour. TBST washes were performed before ECL reagents were applied to the membranes, and radiographic images were captured on X-ray films or by use of the ChemiDoc™ Touch Image System (Bio-Rad, Hercules, CA). GAPDH protein was employed as a loading control.

**High-Performance Liquid Chromatography (HPLC)**

HPLC was utilized to assay KYN and TRP concentrations in cell culture media. 50 μL of cell culture media collected as described above was injected directly into a Shimadzu UFLC system for analysis. HPLC conditions for the assay of KYN and TRP were modified from previously published methods[^9^](#_ENREF_9). A LiChrosorb^®^ RP 18-5 (Supelco, Bellefonte, PA) C18 column (250 mm × 4.6 mm, 5 μm) with a mobile phase consisting of 10 mmol/L acetate buffer (pH 4.5) and acetonitrile (95:5, v/v, 0-10 mins; gradient from 95:5 to 80:20, v/v, 10-16 mins) at a flow rate of 1.0 mL/min at 30 °C was used to assay KYN and TRP. KYN and TRP were monitored at 360 nm and 280 nm, respectively. KYN and TRP concentrations in the cell culture media were calculated based on a standard curve generated by the analysis of KYN and TRP standards (Sigma, St. Louis, MI) at concentrations that ranged from 0.2 to 100 μM and from 1 to 200 μM, respectively.

***SUPPLEMENTARY REFERENCES:***

1. Mrazek DA, Biernacka JM, McAlpine DE, Benitez J, Karpyak VM, Williams MD *et al.* Treatment outcomes of depression: the pharmacogenomic research network antidepressant medication pharmacogenomic study. *J Clin Psychopharmacol* 2014; **34**(3)**:** 313-317.

2. Zhu H, Bogdanov MB, Boyle SH, Matson W, Sharma S, Matson S *et al.* Pharmacometabolomics of response to sertraline and to placebo in major depressive disorder - possible role for methoxyindole pathway. *PLoS One* 2013; **8**(7)**:** e68283.

3. Gupta M, Neavin D, Liu D, Biernacka J, Hall-Flavin D, Bobo WV *et al.* TSPAN5, ERICH3 and selective serotonin reuptake inhibitors in major depressive disorder: pharmacometabolomics-informed pharmacogenomics. *Molecular psychiatry* 2016.

4. Ji Y, Biernacka JM, Hebbring S, Chai Y, Jenkins GD, Batzler A *et al.* Pharmacogenomics of selective serotonin reuptake inhibitor treatment for major depressive disorder: genome-wide associations and functional genomics. *Pharmacogenomics J* 2013; **13**(5)**:** 456-463.

5. Ji Y, Schaid DJ, Desta Z, Kubo M, Batzler AJ, Snyder K *et al.* Citalopram and escitalopram plasma drug and metabolite concentrations: genome-wide associations. *British journal of clinical pharmacology* 2014; **78**(2)**:** 373-383.

6. Lee SH, Jun HK, Lee HR, Chung CP, Choi BK. Antibacterial and lipopolysaccharide (LPS)-neutralising activity of human cationic antimicrobial peptides against periodontopathogens. *Int J Antimicrob Agents* 2010; **35**(2)**:** 138-145.

7. Zwilling D, Huang SY, Sathyasaikumar KV, Notarangelo FM, Guidetti P, Wu HQ *et al.* Kynurenine 3-monooxygenase inhibition in blood ameliorates neurodegeneration. *Cell* 2011; **145**(6)**:** 863-874.

8. Ingle JN, Liu M, Wickerham DL, Schaid DJ, Wang L, Mushiroda T *et al.* Selective estrogen receptor modulators and pharmacogenomic variation in ZNF423 regulation of BRCA1 expression: individualized breast cancer prevention. *Cancer Discov* 2013; **3**(7)**:** 812-825.

9. Zhen Q, Xu B, Ma L, Tian G, Tang X, Ding M. Simultaneous determination of tryptophan, kynurenine and 5-hydroxytryptamine by HPLC: Application in uremic patients undergoing hemodialysis. *Clin Biochem* 2011; **44**(2-3)**:** 226-230.

***SUPPLEMENTARY FIGURES & LEGENDS:***

**Figure S1. *DEFB1*, *AHR* SNPs and Plasma KYN in 290 MDD Patients.** (**A**) The *DEFB1* rs5743467 SNP variant genotype (G/G) was significantly associated with higher baseline plasma KYN concentrations. (**B**) The top *AHR* SNP, rs17137566, variant genotype (C/C) was significantly associated with lower plasma KYN concentrations. Values are mean ± S.D.(**C**) Patients who were homozygous for the WT genotype for the *DEFB1* SNP (rs5743467, C/C) and who were homozygous variant for the *AHR* SNP (rs17137566, C/C) demonstrated associations with lower average plasma KYN concentrations. Moreover, patients who were homozygous variant for the *DEFB1* SNP (rs5743467, G/G) and homozygous WT for the *AHR* SNPs (rs17137566, T/T) displayed associations with the highest average plasma KYN concentrations.


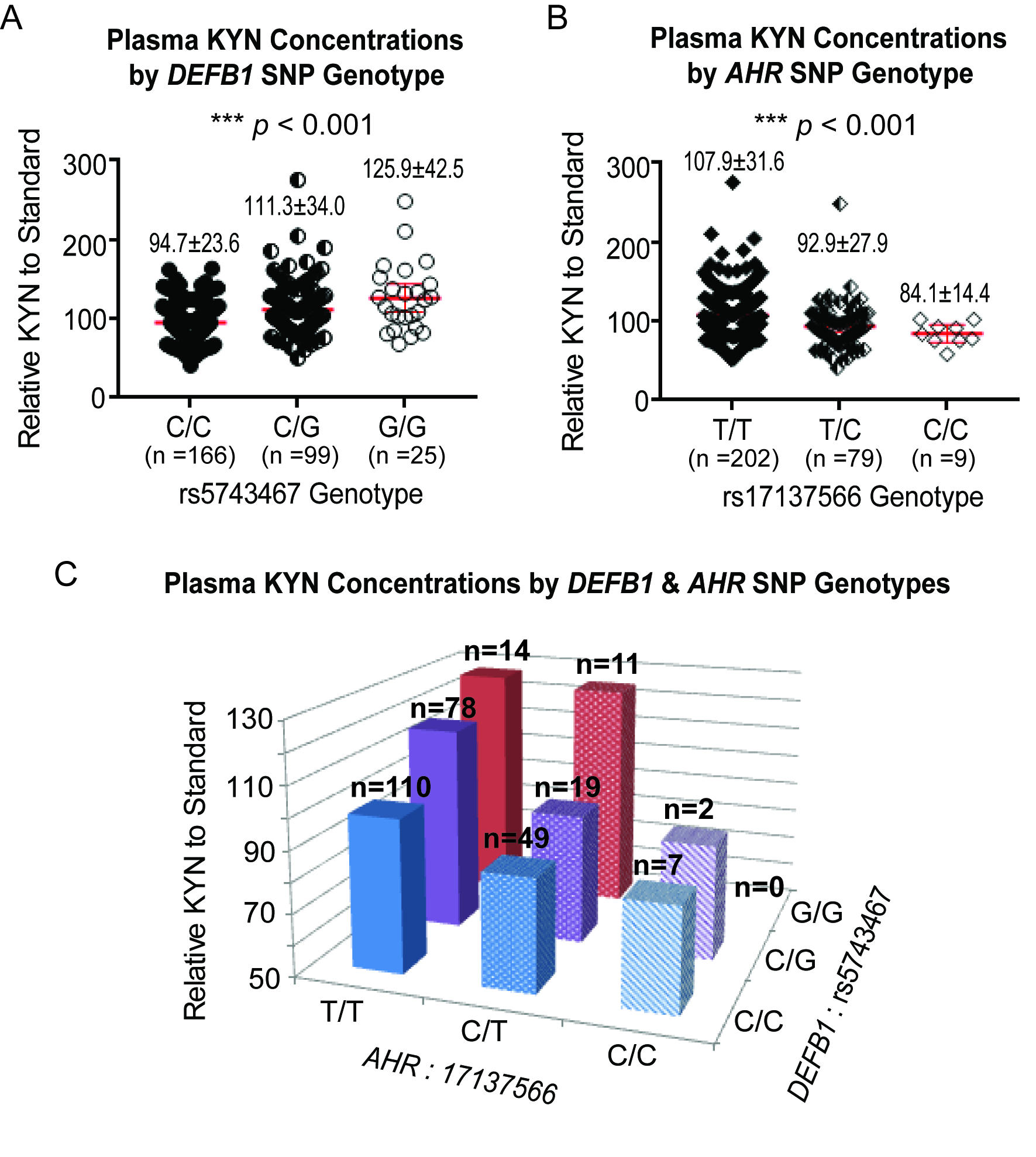


**Figure S2. Plasma K/T Ratio GWAS.** **(A)** Manhattan Plot for baseline plasma K/T ratios. SNPs across the *DEFB1* gene have been highlighted, with rs5743467 as the “top” *DEFB1* SNP (*p* values = 2.15E-07). **(B)** Regional association plots for the *DEFB1* gene. Circles and diamonds represent observed and imputed SNPs, respectively. Colors of the circles and diamonds represent the extent of linkage disequilibrium (LD) with the “top” SNP, which is colored purple.


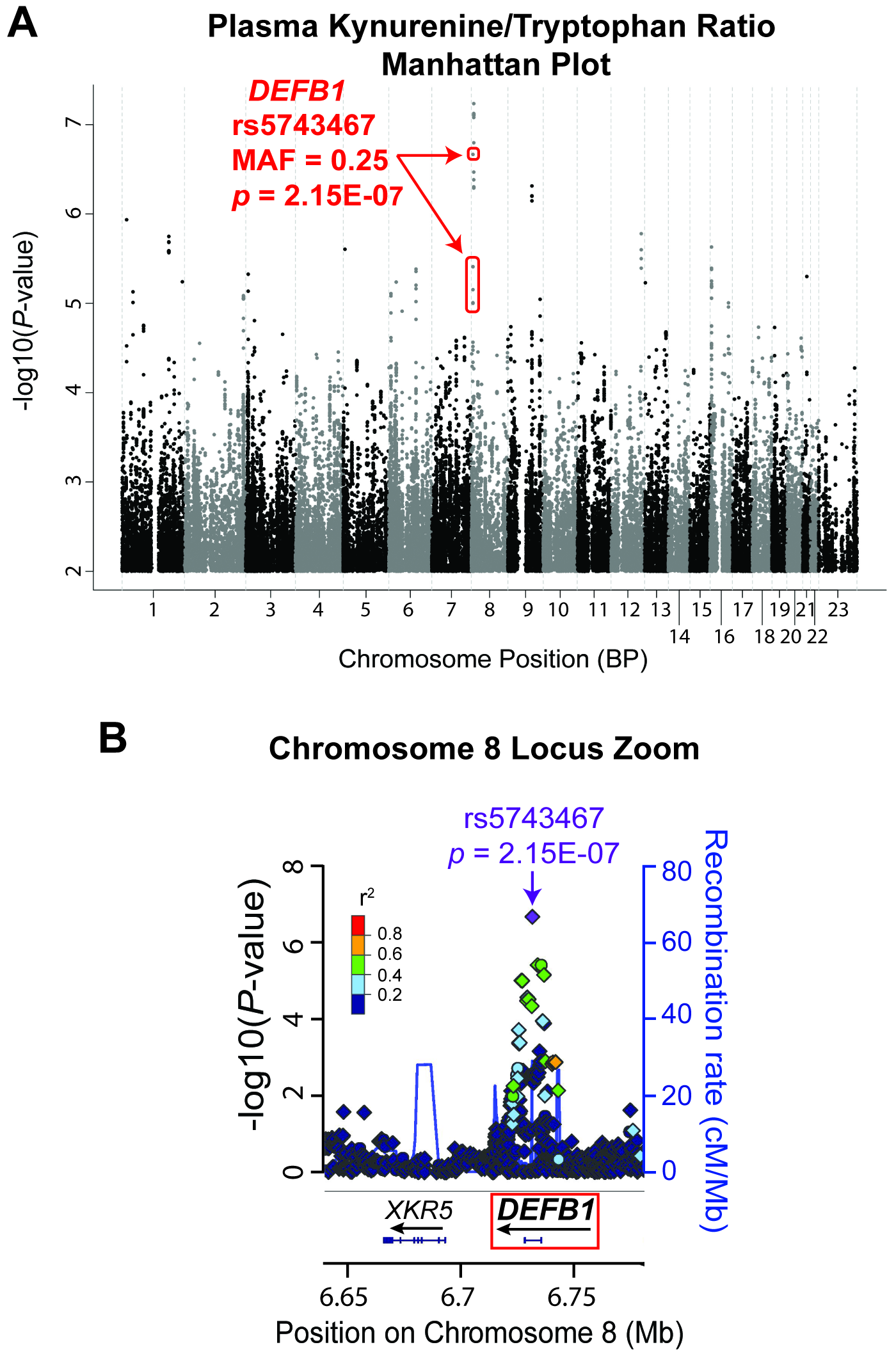


**Figure S3: Kynurenine and Tryptophan Concentrations in HepaRG (A-D) and U-87 MG (E-H) Cell Media that Were Assayed by HPLC.** (**A**) KYN concentrations in the HepaRG cell culture media were significantly decreased after *AHR* KD but were not decreased as greatly when *KMO* or *KYNU* were knocked down together with *AHR*, respectively. (**B**) TRP concentrations were decreased to a similar extent after *AHR* KD and after *AHR* KD plus *KMO* or *KYNU* KD. (**C**) The decrease in KYN concentrations after *AHR* KD was abolished by treatment with the KMO inhibitor Ro 61-8048, but (**D**) TRP concentrations were not significantly altered after incubation with this inhibitor. (**E**) KYN concentrations in U-87 MG cell culture media were significantly decreased after *AHR* KD but were not decreased to the same extent after *KMO* or *KYNU* KD together with *AHR* KD. (**F**) U-87 MG TRP cell culture concentrations were significantly decreased after *AHR* KD as well as after *AHR* KD together with *KMO* or *KYNU* KD. (**G**) KYN concentrations in U-87 MG cell culture media were not significantly decreased after AHR KD together with a KMO inhibitor (Ro 61-8048) as compared with vehicle treatment. (**H**) TRP U-87 MG cell culture media concentrations were not significantly different from vehicle treatment when treated with a KMO inhibitor (Ro 61-8048). N ≥ 3 for all the experiments. Data = mean ± SEM, with statistical significance denoted as, **p* < 0.05, ***p* <0.01, ****p* <0.001 and ns = not significant.

(see **Figure S3** in next page)

**Figure S3:**


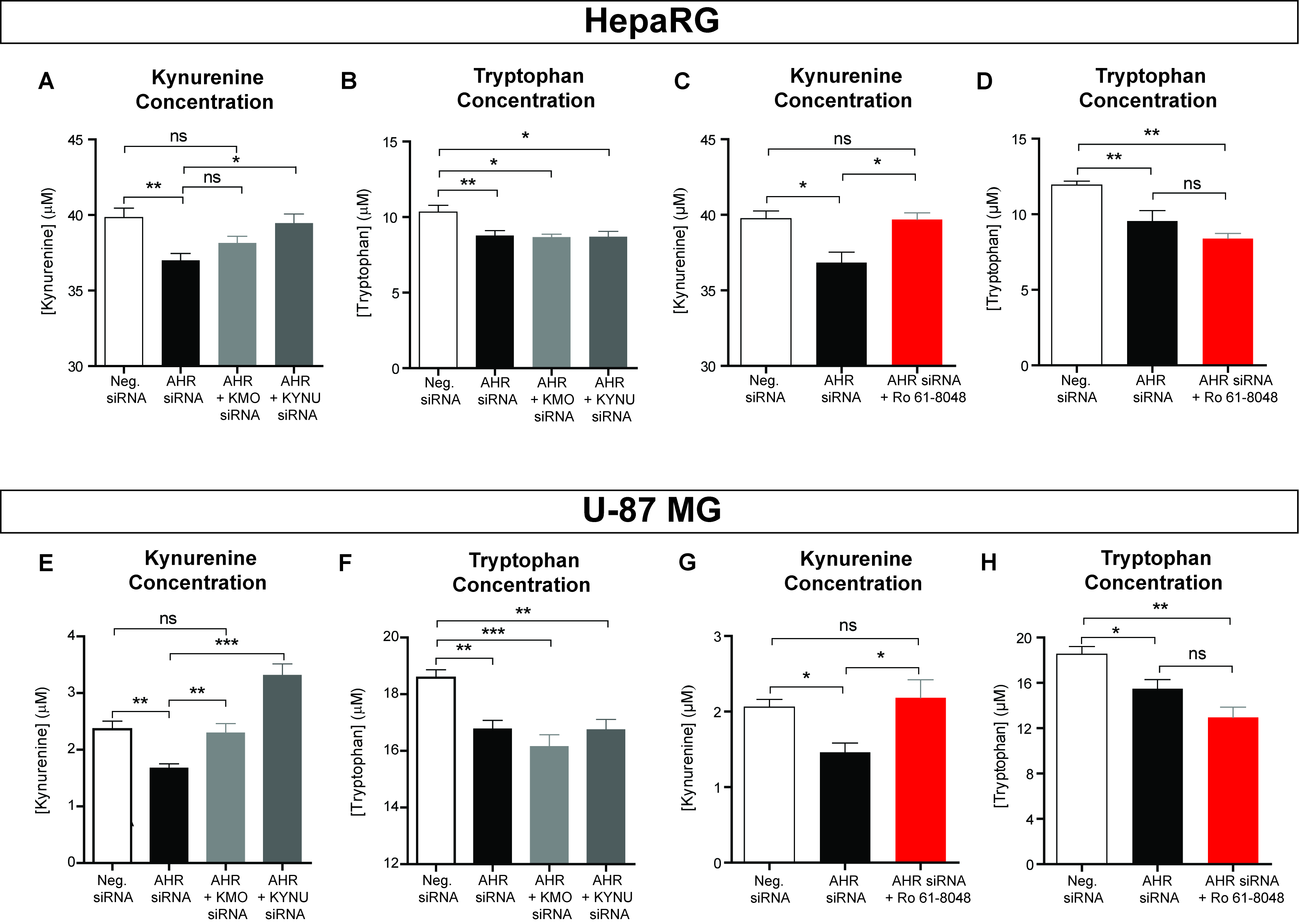


**Figure S4. hNSC-derived Neuronal Cell Functional Studies.** Expression of *AHRR*, *ARNT*, *TDO2*, *KMO* and *KYNU* as measured by qRT-PCR after *AHR* KD. N ≥ 3 for all the experiments. Data = mean ± SEM, with statistical significance determined by two-tailed *t* test denoted as **p* < 0.05, ***p* < 0.01 and ****p* <0.001 when compared with control.


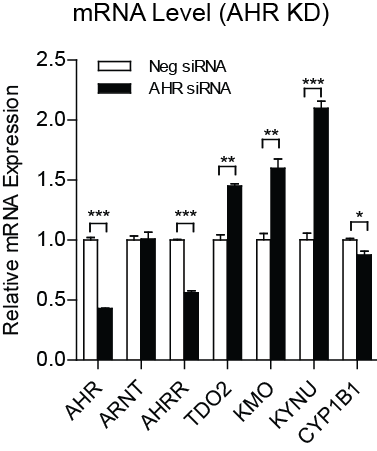


**Figure S5. eQTL Analysis for the *DEFB1* rs2702877 SNP.** *DEFB1* mRNA expression was significantly increased in human cerebellum (left), sigmoid colon (middle) and esophageal muscularis (right) from individuals with rs2702877 variant genotypes (G) when compared with WT genotype (C) based on the GTEx data.

***
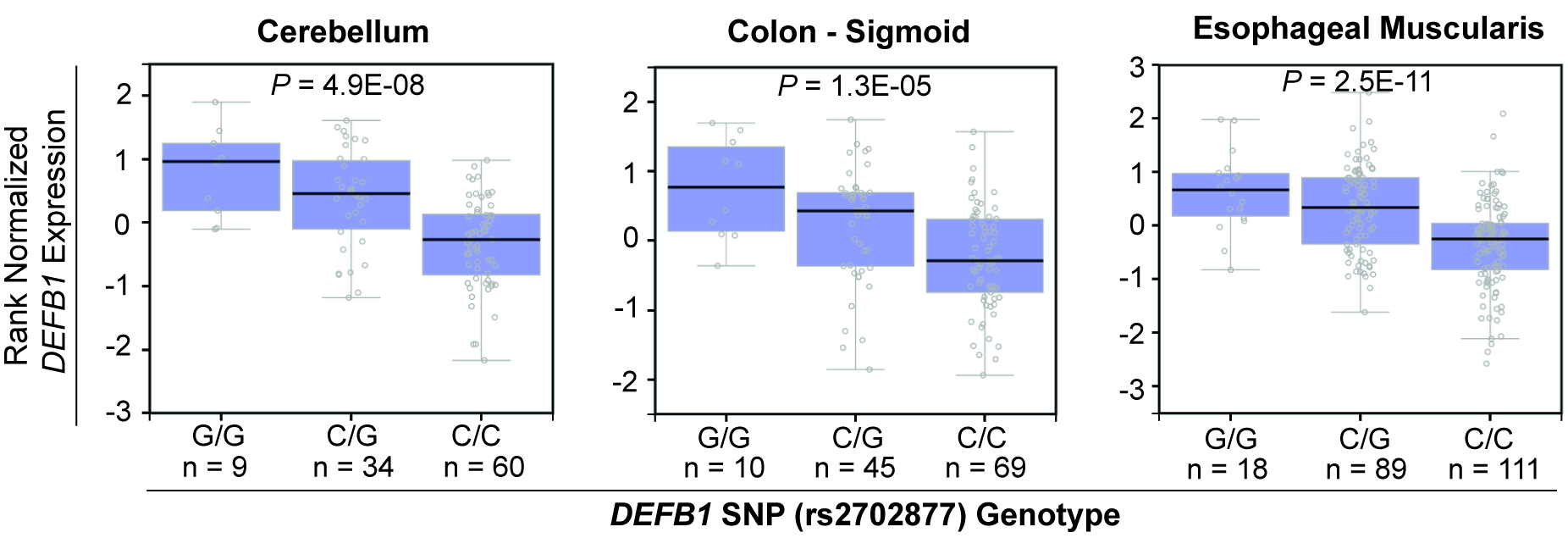
***

***SUPPLEMENTARY TABLES:***

**Table S1. Association of plasma metabolite concentrations with HAMD-17 scores in 290 MDD patients.**

| **Metabolite** | ***r*** | ***p*-value** | **Pathway** |
| --- | --- | --- | --- |
| **Kynurenine** | **-0.157** | **0.008** | **Tryptophan** |
| 3-Hydroxykynurenine | -0.143 | 0.015 | Tryptophan |
| Cysteine | -0.134 | 0.023 | Cysteine |
| Methionine | 0.106 | 0.072 | Methionine |
| Serotonin | -0.099 | 0.093 | Tryptophan |
| Guanosine | 0.099 | 0.095 | Purine |
| 5-Hydroxytrptophan | 0.098 | 0.097 | Tryptophan |
| (+)-delta-Tocopherol | 0.094 | 0.112 | Antioxidants |
| Xanthosine | 0.097 | 0.159 | Purine |
| Salicylic Acid | -0.082 | 0.163 | Phenylalanine |
| 4-Hydroxyphenyllactic acid | 0.077 | 0.192 | Phenylalanine |
| (+)-gamma-Tocopherol | 0.070 | 0.234 | Antioxidants |
| Vanillylmandelic Acid | -0.070 | 0.235 | Tyrosine |
| Tryptophan | 0.069 | 0.245 | Tryptophan |
| AMTRP | -0.064 | 0.279 | Tryptophan |
| Indole-3-propionic acid | -0.059 | 0.319 | Tryptophan |
| 1,7-diMethylxanthine | -0.058 | 0.327 | Purine |
| 1,3-diMethylxanthine | -0.057 | 0.340 | Purine |
| (+)-alpha-Tocopherol | 0.052 | 0.382 | Antioxidants |
| Uric acid | -0.045 | 0.450 | Purine |
| Tyrosine | 0.037 | 0.531 | Tyrosine |
| 4-Hydroxyphenylacetic acid | 0.032 | 0.583 | Tyrosine |
| Indole-3-acetic acid | 0.032 | 0.587 | Tryptophan |
| Hypoxathine | 0.032 | 0.595 | Purine |
| Methoxy-Hydroxyphenly Glycol | -0.031 | 0.597 | Tyrosine |
| Guanine | -0.031 | 0.600 | Purine |
| 5-Hydroxyindoleacetic acid | -0.029 | 0.619 | Tryptophan |
| Homovanillic Acid | -0.025 | 0.671 | Tyrosine |
| 4-Hydroxybenzoic acid | 0.020 | 0.751 | Phenylalanine |
| Xanthine | -0.019 | 0.755 | Purine |
| Homogentisic Acid | 0.002 | 0.980 | Tyrosine |

**Table S2. Top SNPs with lowest p-values for the baseline plasma KYN GWAS.**

| **SNP ID** | **Chr** | **Position^1^** | **Effect Size^2^** | ***P-*value** | **Minor Allele** | **Major Allele** | **MAF** | **Type of SNP^3^** | **Gene** | **Entrez Gene ID** | **Ref Seq ID** | **SNP Distance (bp) from Gene** | **Variant Location** |
| --- | --- | --- | --- | --- | --- | --- | --- | --- | --- | --- | --- | --- | --- |
| rs982422 | 12 | 78847259 | -0.47 | 5.15E-07 | A | C | 0.24 | O |  | 359734 |  | 19835 | 3'downstream |
| **rs5743467** | **8** | **6731529** | **0.43** | **8.18E-07** | **G** | **C** | **0.25** | **I** | **DEFB1** | **1672** | **NM-005218.3** | **0** | **intron** |
| rs61475845 | 11 | 99818976 | -2.2 | 1.24E-06 | A | G | 0.02 | I | CNTN5 | 53942 | NM-014361.2 | 0 | intron |
| rs61475845 | 11 | 99818976 | -2.2 | 1.24E-06 | A | G | 0.02 | I | CNTN5 | 53942 | NM-175566.1 | 0 | intron |
| rs74826345 | 7 | 12198445 | -1.63 | 4.43E-06 | T | G | 0.01 | I | TMEM106B | 54664 | NM-018374.3 | 52403 | 5'upstream |
| rs12113017 | 7 | 12199422 | -1.62 | 4.59E-06 | A | T | 0.01 | I | TMEM106B | 54664 | NM-018374.3 | 51426 | 5'upstream |
| rs2236231 | 14 | 68039027 | -0.4 | 4.74E-06 | A | G | 0.32 | I | PLEKHH1 | 57475 | NM-020715.2 | 0 | intron |
| **rs17137566** | **7** | **17360521** | **-0.47** | **6.22E-06** | **C** | **T** | **0.17** | **I** | **AHR** | **196** | **NM-001621.4** | **0** | **intron** |
| **rs73079677** | **7** | **17365574** | **-0.44** | **6.22E-06** | **G** | **A** | **0.21** | **I** | **AHR** | **196** | **NM-001621.4** | **0** | **intron** |
| rs213862 | 6 | 97234786 | -0.51 | 6.81E-06 | A | G | 0.14 | I | GPR63 | 81491 | NM-030784.2 | 11102 | 3'downstream |
| Abbreviations: Chr. , chromosome; MAF, minor allele frequency; bp, base pairs | | | | | | | | |  |  |  |  |  |
| ^1^ Position was based on the human genome assembly GRCh37. | | | | | | |  |  |  |  |  |  |  |
| ^2^ Average change in transformed KYN concentrations for each additional copy of the SNP minor allele expressed in relative standard deviates (i.e. 2.0=2x31.1; The average KYN concentration was 103.0 ± 31.1 (Mean ± SD). | | | | | | | | | | | | | |
| ^3^ Type of SNP in GWAS, O = observed, I = imputed. | | | | |  |  |  |  |  |  |  |  |  |

**Table S3. Top DEFB1 SNPs for the baseline plasma KYN GWAS with P-values less than 5E-04.**

| **SNP ID** | **Chr** | **Position^1^** | **Effect Size^2^** | ***P-*value** | **Minor Allele** | **Major Allele** | **MAF** | **Type of SNP^3^** | **Gene** | **Entrez Gene ID** | **Ref Seq ID** | **SNP Distance (bp) from Gene** | **Variant Location** |
| --- | --- | --- | --- | --- | --- | --- | --- | --- | --- | --- | --- | --- | --- |
| rs5743467 | 8 | 6731529 | 0.43 | 8.18E-07 | G | C | 0.25 | I | DEFB1 | 1672 | NM-005218.3 | 0 | intron |
| rs5743402 | 8 | 6737285 | 0.41 | 4.03E-05 | C | T | 0.2 | I | DEFB1 | 1672 | NM-005218.3 | 1756 | 5'upstream |
| rs2741130 | 8 | 6733871 | 0.31 | 5.06E-05 | G | A | 0.43 | I | DEFB1 | 1672 | NM-005218.3 | 0 | intron |
| rs11362 | 8 | 6735399 | 0.31 | 5.18E-05 | A | G | 0.43 | O | DEFB1 | 1672 | NM-005218.3 | 0 | 5'UTR |
| rs2702877 | 8 | 6736067 | -0.34 | 7.87E-05 | G | C | 0.31 | I | DEFB1 | 1672 | NM-005218.3 | 538 | 5'upstream |
| rs5743409 | 8 | 6736620 | 0.3 | 9.05E-05 | A | C | 0.43 | I | DEFB1 | 1672 | NM-005218.3 | 1091 | 5'upstream |
| rs2741121 | 8 | 6727082 | 0.3 | 1.20E-04 | T | C | 0.43 | I | DEFB1 | 1672 | NM-005218.3 | 1015 | 3'downstream |
| rs2741120 | 8 | 6726700 | 0.3 | 1.21E-04 | C | T | 0.43 | I | DEFB1 | 1672 | NM-005218.3 | 1397 | 3'downstream |
| rs5743482 | 8 | 6729918 | 0.29 | 1.75E-04 | A | G | 0.44 | I | DEFB1 | 1672 | NM-005218.3 | 0 | intron |
| rs2738173 | 8 | 6740938 | 0.37 | 1.79E-04 | G | A | 0.19 | O | DEFB1 | 1672 | NM-005218.3 | 5409 | 5'upstream |
| rs73188256 | 8 | 6740618 | 0.37 | 1.85E-04 | C | T | 0.19 | I | DEFB1 | 1672 | NM-005218.3 | 5089 | 5'upstream |
| rs10095283 | 8 | 6741662 | 0.37 | 1.88E-04 | A | G | 0.18 | I | DEFB1 | 1672 | NM-005218.3 | 6133 | 5'upstream |
| rs2951852 | 8 | 6741807 | 0.37 | 1.90E-04 | A | G | 0.18 | I | DEFB1 | 1672 | NM-005218.3 | 6278 | 5'upstream |
| rs2741125 | 8 | 6729122 | 0.29 | 2.00E-04 | G | A | 0.44 | I | DEFB1 | 1672 | NM-005218.3 | 0 | intron |
| rs73188255 | 8 | 6740187 | 0.38 | 2.01E-04 | A | G | 0.18 | I | DEFB1 | 1672 | NM-005218.3 | 4658 | 5'upstream |
| rs2741126 | 8 | 6731280 | 0.28 | 2.15E-04 | T | C | 0.44 | I | DEFB1 | 1672 | NM-005218.3 | 0 | intron |
| rs2741112 | 8 | 6725517 | 0.28 | 2.62E-04 | G | C | 0.49 | I | DEFB1 | 1672 | NM-005218.3 | 2580 | 3'downstream |
| Abbreviations: Chr. , chromosome; MAF, minor allele frequency; bp, base pairs | | | | | | | |  |  |  |  |  |  |
| ^1^ Position was based on the human genome assembly GRCh37. | | | | | |  |  |  |  |  |  |  |  |
| ^2^ Average change in transformed KYN concentrations for each additional copy of the SNP minor allele expressed in relative standard deviates (i.e. 2.0=2x31.1; The average KYN concentration was 103.0 ± 31.1 (Mean ± SD). | | | | | | | | | | | | | |
| ^3^ Type of SNP in GWAS, O = observed, I = imputed. | | | | |  |  |  |  |  |  |  |  |  |

**Table S4. Top *AHR* SNPs for the baseline plasma KYN GWAS with P-values less than 1E-04.**

| **SNP ID** | **Chr** | **Position^1^** | **Effect Size^2^** | ***P-*value** | **Minor Allele** | **Major Allele** | **MAF** | **Type of SNP^3^** | **Gene** | **Entrez Gene ID** | **Ref Seq ID** | **SNP Distance (bp) from Gene** | **Variant Location** |
| --- | --- | --- | --- | --- | --- | --- | --- | --- | --- | --- | --- | --- | --- |
| rs17137566 | 7 | 17360521 | -0.47 | 6.22E-06 | C | T | 0.17 | I | AHR | 196 | NM-001621.4 | 0 | intron |
| rs73079677 | 7 | 17365574 | -0.44 | 6.22E-06 | G | A | 0.21 | I | AHR | 196 | NM-001621.4 | 0 | intron |
| rs3802082 | 7 | 17370181 | -0.46 | 7.52E-06 | T | A | 0.17 | I | AHR | 196 | NM-001621.4 | 0 | intron |
| rs10272066 | 7 | 17374902 | -0.42 | 8.68E-06 | C | T | 0.21 | I | AHR | 196 | NM-001621.4 | 0 | intron |
| rs2053998 | 7 | 17407061 | -0.4 | 1.19E-05 | G | A | 0.22 | I | AHR | 196 | NM-001621.4 | 21286 | 3'downstream |
| rs7791070 | 7 | 17401027 | -0.4 | 1.24E-05 | G | A | 0.22 | O | AHR | 196 | NM-001621.4 | 15252 | 3'downstream |
| rs2078982 | 7 | 17392749 | -0.4 | 1.36E-05 | C | T | 0.22 | I | AHR | 196 | NM-001621.4 | 6974 | 3'downstream |
| rs116442126 | 7 | 17391593 | -0.4 | 1.39E-05 | C | T | 0.22 | I | AHR | 196 | NM-001621.4 | 5818 | 3'downstream |
| rs2040623 | 7 | 17380662 | -0.4 | 1.47E-05 | C | A | 0.22 | O | AHR | 196 | NM-001621.4 | 0 | intron |
| rs4143452 | 7 | 17410930 | -0.39 | 1.68E-05 | C | T | 0.22 | I | AHR | 196 | NM-001621.4 | 25155 | 3'downstream |
| rs7780687 | 7 | 17405718 | -0.42 | 1.79E-05 | G | A | 0.18 | O | AHR | 196 | NM-001621.4 | 19943 | 3'downstream |
| rs10269143 | 7 | 17398983 | -0.43 | 1.82E-05 | A | T | 0.17 | I | AHR | 196 | NM-001621.4 | 13208 | 3'downstream |
| rs12667669 | 7 | 17425895 | -0.38 | 3.74E-05 | G | A | 0.23 | O | AHR | 196 | NM-001621.4 | 40120 | 3'downstream |
| rs2198497 | 7 | 17414133 | -0.39 | 7.76E-05 | G | A | 0.17 | I | AHR | 196 | NM-001621.4 | 28358 | 3'downstream |
| rs1111544 | 7 | 17414784 | -0.39 | 7.88E-05 | G | A | 0.17 | I | AHR | 196 | NM-001621.4 | 29009 | 3'downstream |
| rs9655142 | 7 | 17415828 | -0.39 | 8.63E-05 | A | T | 0.17 | I | AHR | 196 | NM-001621.4 | 30053 | 3'downstream |
| rs10233705 | 7 | 17416616 | -0.39 | 8.67E-05 | G | A | 0.17 | I | AHR | 196 | NM-001621.4 | 30841 | 3'downstream |
| rs2237297 | 7 | 17359594 | -0.49 | 8.74E-05 | A | G | 0.1 | I | AHR | 196 | NM-001621.4 | 0 | intron |
| Abbreviations: Chr. , chromosome; MAF, minor allele frequency; bp, base pairs | | | | | | | |  |  |  |  |  |  |
| ^1^ Position was based on the human genome assembly GRCh37. | | | | | |  |  |  |  |  |  |  |  |
| ^2^ Average change in transformed KYN concentrations for each additional copy of the SNP minor allele expressed in relative standard deviates (i.e. 2.0=2x31.1; The average KYN concentration was 103.0 ± 31.1 (Mean ± SD). | | | | | | | | | | | | | |
| ^3^ Type of SNP in GWAS, O = observed, I = imputed. | | | | |  |  |  |  |  |  |  |  |  |

**Table S5. Top *DEFB1* SNPs for the baseline plasma TRP-to-KYN (K/T) ratio GWAS with p-values less than 5E-04.**

| **SNP ID** | **Chr** | **Position^1^** | **Effect Size^2^** | ***p-*value** | **Minor Allele** | **Major Allele** | **MAF** | **Type of SNP^3^** | **Gene** | **Entrez Gene ID** | **Ref Seq ID** | **SNP Distance (bp) from Gene** | **Variant Location** |
| --- | --- | --- | --- | --- | --- | --- | --- | --- | --- | --- | --- | --- | --- |
| rs5743467 | 8 | 6731529 | 0.46 | 2.15E-07 | G | C | 0.25 | I | DEFB1 | 1672 | NM-005218.3 | 0 | intron |
| rs2741130 | 8 | 6733871 | 0.36 | 3.89E-06 | G | A | 0.43 | I | DEFB1 | 1672 | NM-005218.3 | 0 | intron |
| rs11362 | 8 | 6735399 | 0.36 | 3.89E-06 | A | G | 0.43 | O | DEFB1 | 1672 | NM-005218.3 | 0 | 5'UTR |
| rs5743409 | 8 | 6736620 | 0.35 | 7.03E-06 | A | C | 0.43 | I | DEFB1 | 1672 | NM-005218.3 | 1091 | 5'upstream |
| rs2741120 | 8 | 6726700 | 0.35 | 9.85E-06 | C | T | 0.43 | I | DEFB1 | 1672 | NM-005218.3 | 1397 | 3'downstream |
| rs2741121 | 8 | 6727082 | 0.35 | 9.99E-06 | T | C | 0.43 | I | DEFB1 | 1672 | NM-005218.3 | 1015 | 3'downstream |
| rs79559910 | 8 | 6729314 | 0.73 | 2.73E-05 | A | G | 0.08 | I | DEFB1 | 1672 | NM-005218.3 | 0 | intron |
| rs5743482 | 8 | 6729918 | 0.33 | 3.04E-05 | A | G | 0.44 | I | DEFB1 | 1672 | NM-005218.3 | 0 | intron |
| rs2741125 | 8 | 6729122 | 0.33 | 3.34E-05 | G | A | 0.44 | I | DEFB1 | 1672 | NM-005218.3 | 0 | intron |
| rs2741126 | 8 | 6731280 | 0.32 | 4.57E-05 | T | C | 0.44 | I | DEFB1 | 1672 | NM-005218.3 | 0 | intron |
| rs2702877 | 8 | 6736067 | -0.34 | 1.12E-04 | G | C | 0.31 | I | DEFB1 | 1672 | NM-005218.3 | 538 | 5'upstream |
| rs151113154 | 8 | 6736771 | 0.97 | 1.29E-04 | A | G | 0.04 | I | DEFB1 | 1672 | NM-005218.3 | 1242 | 5'upstream |
| rs2741112 | 8 | 6725517 | 0.29 | 1.92E-04 | G | C | 0.49 | I | DEFB1 | 1672 | NM-005218.3 | 2580 | 3'downstream |
| rs2741113 | 8 | 6725674 | 0.28 | 4.08E-04 | C | G | 0.49 | I | DEFB1 | 1672 | NM-005218.3 | 2423 | 3'downstream |
| rs2741117 | 8 | 6725969 | 0.28 | 4.13E-04 | G | T | 0.49 | I | DEFB1 | 1672 | NM-005218.3 | 2128 | 3'downstream |
| rs2741114 | 8 | 6725681 | 0.27 | 4.36E-04 | G | A | 0.49 | I | DEFB1 | 1672 | NM-005218.3 | 2416 | 3'downstream |
| Abbreviations: Chr. , chromosome; MAF, minor allele frequency; bp, base pairs | | | | | | | | |  |  |  |  |  |
| ^1^ Position was based on the human genome assembly GRCh37. | | | | | | |  |  |  |  |  |  |  |
| ^2^ Average change in transformed KYN concentrations for each additional copy of the SNP minor allele expressed in relative standard deviates (i.e. 2.0=2x31.1; The average KYN concentration was 103.0 ± 31.1 (Mean ± SD). | | | | | | | | | | | | | |
| ^3^ Type of SNP in GWAS, O = observed, I = imputed. | | | | |  |  |  |  |  |  |  |  |  |

**Table S6. Top SNPs for the baseline plasma TRP-to-KYN (K/T) ratio GWAS with p-values less than 10E-07.**

| **SNP ID** | **Chr** | **Position^1^** | **Effect Size^2^** | ***P-*value** | **Minor Allele** | **Major Allele** | **MAF** | **Type of SNP^3^** | **Gene** | **Entrez Gene ID** | **Ref Seq ID** | **SNP Distance (bp) from Gene** | **Variant Location** |
| --- | --- | --- | --- | --- | --- | --- | --- | --- | --- | --- | --- | --- | --- |
| rs75921243 | 8 | 9845866 | -1.51 | 5.82E-08 | G | T | 0.02 | I | MSRA | 4482 | NM-012331.3 | 65964 | 5'upstream |
| rs79314841 | 8 | 9836818 | -1.49 | 7.50E-08 | A | G | 0.02 | I | MSRA | 4482 | NM-012331.3 | 75012 | 5'upstream |
| rs494997 | 8 | 9823186 | -1.43 | 7.62E-08 | G | A | 0.02 | I | MIR124-1 | 406907 | NR-029668.1 | 62204 | 5'upstream |
| rs471930 | 8 | 9823373 | -1.43 | 7.77E-08 | C | T | 0.02 | I | MIR124-1 | 406907 | NR-029668.1 | 62391 | 5'upstream |
| rs580415 | 8 | 9823575 | -1.43 | 7.77E-08 | A | T | 0.02 | I | MIR124-1 | 406907 | NR-029668.1 | 62593 | 5'upstream |
| rs553191 | 8 | 9824244 | -1.43 | 7.95E-08 | T | C | 0.02 | I | MIR124-1 | 406907 | NR-029668.1 | 63262 | 5'upstream |
| rs668151 | 8 | 9822323 | -1.43 | 8.31E-08 | A | G | 0.02 | I | MIR124-1 | 406907 | NR-029668.1 | 61341 | 5'upstream |
| rs75459170 | 8 | 9841829 | -1.52 | 1.59E-07 | G | A | 0.02 | I | MSRA | 4482 | NM-012331.3 | 70001 | 5'upstream |
| **rs5743467** | **8** | **6731529** | **0.46** | **2.15E-07** | **G** | **C** | **0.25** | **I** | **DEFB1** | **1672** | **NM-005218.3** | **0** | **intron** |
| rs146857475 | 8 | 9831588 | -1.39 | 3.39E-07 | G | A | 0.02 | I | MIR124-1 | 406907 | NR-029668.1 | 70606 | 5'upstream |
| rs79263965 | 8 | 9830993 | -1.37 | 4.14E-07 | G | A | 0.02 | I | MIR124-1 | 406907 | NR-029668.1 | 70011 | 5'upstream |
| rs116609693 | 9 | 95573676 | -1.35 | 4.86E-07 | T | C | 0.03 | I | ANKRD19P | 138649 | NR-026868.1 | 0 | intron |
| rs117873532 | 8 | 9828353 | -1.35 | 4.97E-07 | G | C | 0.02 | I | MIR124-1 | 406907 | NR-029668.1 | 67371 | 5'upstream |
| rs80322361 | 8 | 9818104 | -1.35 | 4.97E-07 | T | C | 0.02 | I | MIR124-1 | 406907 | NR-029668.1 | 57122 | 5'upstream |
| rs7820910 | 8 | 9826340 | -1.35 | 5.10E-07 | G | A | 0.02 | O | MIR124-1 | 406907 | NR-029668.1 | 65358 | 5'upstream |
| rs79827930 | 8 | 9823493 | -1.35 | 5.12E-07 | T | C | 0.02 | I | MIR124-1 | 406907 | NR-029668.1 | 62511 | 5'upstream |
| rs112451981 | 9 | 95575089 | -1.33 | 6.26E-07 | A | G | 0.03 | I | ANKRD19P | 138649 | NR-026868.1 | 0 | intron |
| rs113452777 | 9 | 95574648 | -1.33 | 6.30E-07 | T | C | 0.03 | I | ANKRD19P | 138649 | NR-026868.1 | 0 | intron |
| rs7035996 | 9 | 95576800 | -1.33 | 6.30E-07 | T | G | 0.03 | I | ANKRD19P | 138649 | NR-026868.1 | 0 | intron |
| rs116748805 | 9 | 95572194 | -1.33 | 6.30E-07 | T | C | 0.03 | I | ANKRD19P | 138649 | NR-026868.1 | 0 | intron |
| rs116067709 | 9 | 95575722 | -1.33 | 6.32E-07 | G | A | 0.03 | I | ANKRD19P | 138649 | NR-026868.1 | 0 | intron |
| rs7026366 | 9 | 95577926 | -1.18 | 7.13E-07 | G | A | 0.04 | I | ANKRD19P | 138649 | NR-026868.1 | 0 | intron |
| Abbreviations: Chr. , chromosome; MAF, minor allele frequency; bp, base pairs | | | | | | | | |  |  |  |  |  |
| ^1^ Position was based on the human genome assembly GRCh37. | | | | | | |  |  |  |  |  |  |  |
| ^2^ Average change in transformed KYN concentrations for each additional copy of the SNP minor allele expressed in relative standard deviates (i.e. 2.0=2x31.1; The average KYN concentration was 103.0 ± 31.1 (Mean ± SD). | | | | | | | | | | | | | |
| ^3^ Type of SNP in GWAS, O = observed, I = imputed. | | | | |  |  |  |  |  |  |  |  |  |

**Table S7. DEFB1 and AHR SNPs are cis-eQTLs in Brain Tissues (BRAINEAC Datasets).**

| **SNP** | **Expression Probe ID^1^** | **Gene** | **Brain Tissue** | **N** | ***p*-Value** | **Association Direction** |
| --- | --- | --- | --- | --- | --- | --- |
| rs5743467 | 3122692 | DEFB1 | Cerebellar Cortex | 130 | 1.90E-04 | Negative |
| rs5743467 | 3122692 | DEFB1 | Occipital Cortex | 129 | 2.10E-03 | Negative |
| rs5743467 | 3122692 | DEFB1 | Thalamus | 124 | 4.20E-02 | Negative |
| rs5743467 | 3122688 | DEFB1 | Hippocampus | 122 | 3.90E-02 | Negative |
| rs17137566 | 2991235 | AHR | Cerebellar Cortex | 130 | 7.00E-03 | Negative |
| rs17137566 | 2991254 | AHR | Thalamus | 124 | 6.00E-03 | Negative |
| rs17137566 | 2991255 | AHR | Substantia Nigra | 101 | 4.00E-02 | Negative |
| ^1^ID for the probes in the Affymetrix Human Exon 1.0 ST arrays which has been used for mRNA quantification in the BRAINEAC datasets. | | | | | | |

**Table S8. Association of DEFB1 SNP with Severity of MDD Symptoms in Patients Recruited in the Mayo-PGRN AMPS (n = 803).**

|  | | HAMD-17 | | QIDS-C16 | |
| --- | --- | --- | --- | --- | --- |
| SNP | Gene | *p-*Value | Beta | *p-*Value | Beta |
| rs2702877 | DEFB1 | 1.74E-04 | 0.9422 | 1.25E-05 | 1.5987 |

**Table S9. Primary Antibodies Used to Perform Western Blot Analyses**

| Antibody | Company | Catalog Number |
| --- | --- | --- |
| AHR | Cell Signaling | 13790S |
| IDO1 | Sigma | HPA023072 |
| TDO2 | Abnova | H00008942-B01 |
| KMO | abcom | ab93195 |
| KYNU | GeneTex | GTX115169 |
| GAPDH | Novus | NB300-221 |
